# Domain architecture of the *Mycobacterium tuberculosis* MabR (*Rv2242*), a member of the PucR transcription factor family

**DOI:** 10.1016/j.heliyon.2024.e40494

**Published:** 2024-11-16

**Authors:** Véronique Megalizzi, Abdalkarim Tanina, Camille Grosse, Manon Mirgaux, Pierre Legrand, Gaëtan Dias Mirandela, Alexandre Wohlkönig, Pablo Bifani, René Wintjens

**Affiliations:** a Unit of Microbiology, Bioorganic and Macromolecular Chemistry, Department of Research in Drug Development, Faculty of Pharmacy, Université Libre de Bruxelles, Belgium; b Laboratoire de Chimie Biologique Structurale (CBS), Unité de Chimie Physique Théorique et Structurale (UCPTS), Department of Chemistry, Faculty of Sciences, University of Namur, Belgium; c Center of Microscopy and Molecular Imaging (CMMI), Biopark Charleroi, Université Libre de Bruxelles, Gosselies, Belgium; d SOLEIL synchrotron, Gif-sur-Yvette, France; e Biology of Membrane Transport Laboratory, Molecular Biology Department, Faculty of Sciences, Université Libre de Bruxelles, Belgium; f Center for Structural Biology, Vlaams Institute voor Biotechnology (VIB), Brussels, Belgium; g A∗STAR Infectious Diseases Laboratory, Agency for Science, Technology and Research (A∗STAR), Singapore; h Lee Kong Chian School of Medicine, Nanyang Technological University, Singapore

**Keywords:** FAS-II, Mycolic acids, Oligomerization, DNA-Binding, AlphaFold, X-ray crystallography, Electron microscopy, Gel filtration, SAXS

## Abstract

MabR (*Rv2242*), a PucR-type transcription factor, plays a crucial role in regulating mycolic acid biosynthesis in *Mycobacterium tuberculosis*. To understand its regulatory mechanisms, we determined the crystal structures of its N-terminal and C-terminal domains. The N-terminal domain adopts a globin-like fold, while the C-terminal domain comprises an α/β GGDEF domain and an all-α effector domain with a helix-turn-helix DNA-binding motif. This unique domain combination is specific to *Actinomycetes*. Biochemical and computational studies suggest that full-length MabR forms both dimeric and tetrameric assemblies in solution. Structural analysis revealed two distinct dimerization interfaces within the N- and C-terminal domains, further supporting a tetrameric organization. These findings provide valuable insights into the domain architecture, oligomeric state, and potential regulatory mechanisms of MabR.

## 1 Introduction

Tuberculosis (TB) remains a global health challenge, with over 10 million cases and 1.3 million deaths in 2022 [1]. In 2020–21, TB was the second leading cause of death from a single infectious agent, ranking below SARS-CoV-2 but above HIV/AIDS. The increasing prevalence of mycobacterial strains resistant to first-line treatments poses a major obstacle to TB control efforts. In 2022, an estimated 410,000 cases were multidrug-resistant (MDR-TB) or rifampicin-resistant., and out of 2.9 million tested TB cases, 5.1 % were detected as MDR-TB, while 1 % were identified as extensively drug-resistant (XDR-TB) [1]. This alarming situation highlights the urgent need to identify new mycobacterial targets for developing novel TB therapies.

A distinctive feature of mycobacteria is the unusual composition of their cell wall, where mycolic acids (MAs) serve as specific and critical structural components [2]. MAs contribute to the permeability of the bacterial cell envelope and are essential for both viability and virulence, making the proteins involved in mycolate biosynthesis prime drug targets [3]. Consequently, many existing TB drugs, such as isoniazid and ethionamide, as well as several drugs in development, target MA biosynthesis [4].

MA biosynthesis involves two distinct pathways: the fatty acid synthase types I (FAS-I) and II (FAS-II). FAS-I is a multifunctional enzyme that contains all protein domains necessary for the *de novo* synthesis of short-chain fatty acids C_16_-C_18_, which can either form the saturated α-branch C_24_ or be extended by FAS-II to generate the verylong meromycolate chain [5,6]. In *Mycobacterium tuberculosis* (*Mtb*), FAS-I is encoded by a unique gene (H37Rv strain, *Rv2524c*), while the components of FAS-II are spread across three operons. These include the *fas-II* operon, containing the *fabD*, *acpM*, *kasA*, *kasB*, and *accD6* genes (*Rv2243-47*); the *fas-II* reductase operon, with *mabA*/*fabG1* and *inhA*; and the *fas-II* dehydratase operon, which includes the *hadA*, *hadB*, and *hadC* genes (*Rv0635-37*) [7,8].

Regulation of the *fas-II* operon is mediated by *Rv2242*, the gene product located immediately upstream of the operon promoter. Its binding to the *fas-II* promoter region has been confirmed both *in vitro* and *in vivo* [9]. This gene product is known as MabR (Mycolic acid biosynthesis Regulator). Initially, MabR was described as a repressor based on overexpression and knockdown studies of its homolog in *M. smegmatis* (*MSMEG_4324*) [9]. Additionally, it was observed that overexpression of SWU1gp39, a gene from the mycobacteriophage SWU1 with unknown function, increased the expression level of *MSMEG_4324* the homolog of *Rv2242*, by more than 128-fold in *M. smegmatis*, leading to repression of the primary *fas-II* operon [10].

However, a more recent study using a tetracycline-inducible system revealed that sub-physiological levels of MabR resulted in downregulation of the *fas-II* operon in *M. smegmatis*, suggesting a potential role for MabR as a transcriptional activator of *fas-II* [11]. This highlights the need for further investigation into the direct transcriptional influence of MabR on the *fas-II* operon. It is also worth noting that research on MabR remains limited, with little information available regarding the MabR protein.

In this study, we report the *in vitro* structural characterization of the *Mtb* MabR protein, a member of the PucR family of transcriptional factors. The crystal structures of its N- and C-terminal fragments were determined at resolutions of 3.6 Å and 2.0 Å, respectively. The N-terminal region adopts a nonheme globin sensor domain, while the C-terminal region contains a GGDEF-like domain paired with a Helix-Turn-Helix (HTH) motif. This domain combination appears to be unique to *Actinomycetes*. In solution, the full-length MabR exists in both dimeric and tetrameric forms. *AlphaFold* models of the full-length MabR in its dimeric and tetrameric forms reveal two distinct dimeric interfaces corresponding to those observed in the crystal structures of the isolated N- and C- terminal domains. This is the first study to present the complete structure of a PucR-type transcription factor, providing novel insights into the structural organization and oligomerization mechanisms of this regulatory family.

## 2 Results

### 2.1 N-MabR adopts an all-α structure

The crystal structure of N-MabR was solved using the molecular replacement method with an *AlphaFold2* (*AF2*) predicted model as a template and refined to a resolution of 3.6 Å. Data collection and refinement statistics are provided in Table 1. The crystal asymmetric unit contains four superimposable N-MabR molecules with a mean root-mean-square deviation (rmsd) of 1.8 Å, calculated over approximately 140 aligned Cα atoms. The segment spanning Ser^83^ to Leu^105^ appeared highly disordered in the crystal and could only be modeled as a coil in each chain.

**Table 1:** Data collection and refinement statistics.

	N-MabR	C-MabR Hg-derivative	C-MabR Native
**Data Collection**^(^a^)^			
PDB ID	9FB1		9F80
Synchrotron beamline	SOLEIL-PX1	SOLEIL-PX2A	SOLEIL-PX1
Wavelength (Å)	0.9786	1.0064	0.9786
Space group	*P*4_1_22	*C*222_1_	*C*222_1_
Unit cell parameters (Å)	107.19; 107.19; 176.51	63.91; 75.39; 139.72	63.19; 75.41; 139.93
Resolution range (Å)	57.50–3.59 (3.76–3.59)	48.90–2.45 (2.59–2.45)	48.38–2.03 (2.15–2.03)
Spherical completeness (%)	87.3 (36.0)	99.7 (98.0)	99.7 (98.5)
Ellipsoidal completeness^(^b^)^ (%)	95.0 (76.5)	–	–
No. of reflections	287962 (13972)	164267 (25263)	293515 (46568)
No. of unique reflections	10989 (549)	24260 (3853)	21908 (3440)
Multiplicity	26.2 (24.4)	6.8 (6.5)	13.4 (13.5)
R_meas_^(^c^)^	0.172 (1.660)	0.640 (0.992)	0.073 (1.353)
<I/σ(I)>^(^d^)^	11.3 (2.3)	17.14 (1.65)	22.81 (2.01)
CC_1/2_^(^e^)^	0.999 (0.822)	0.937 (0.636)	0.732 (0.681)
**Refinement statistics**			
Number of reflections^(^f^)^	10454 (251)	–	20812 (1096)
Number of refined atoms		–	
Protein	4575	–	1787
Water	0	–	38
Other	0	–	9
Final R_work_^(^g^)/^R_free_^(^h^)^ (%)	25.0/32.5	–	19.7/25.4
RMSD bond lengths (Å)	0.006	–	0.015
RMSD bond angles (°)	1.832	–	2.206
Overall mean B factor (Å^2^)	161.35	–	51.79
**MolProbity statistics**			
Ramachandran favored (%)	93.92	–	97.49
Ramachandran outliers (%)	0.0	–	0.0
Clash score all-atom	24.36 (89th percentile)	–	2.79 (99th percentile)
MolProbity score	2.95 (90th percentile)	–	1.43 (98th percentile)

a For data-collection, the numbers in parentheses represent values for the highest resolution shell.

b Ellipsoidal completeness was obtained from anisotropic data analysis with STARANISO [12].

c R_meas_ = ∑_hkl_ [*N*/(*N* -1)] ^½^ ∑_i_ |I_i_(hkl) - <I(hkl)>|/∑_hkl_ ∑_i_ I_i_(hkl), is the multiplicity (*N*) independent R_merge_, where I_i_ is the intensity of the ith observation and <I> is the mean intensity of the reflections [13].

d <I/σ(I)> = mean of I/σ(I) of unique reflections.

e The mean intensity correlation coefficient of half-datasets [14].

f Numbers in parentheses are the number of reflections in the free set.

g R_work_ = ∑||F_obs_| - |F_calc_||/∑|F_obs_| , where F_calc_ and F_obs_ are the calculated and observed structure factor amplitude, respectively.

h R_free_ = ∑||F_obs_| - |F_calc_||/∑|F_obs_| , where all reflections belong to a test set of 5% randomly selected data.

**Fig. 1:**
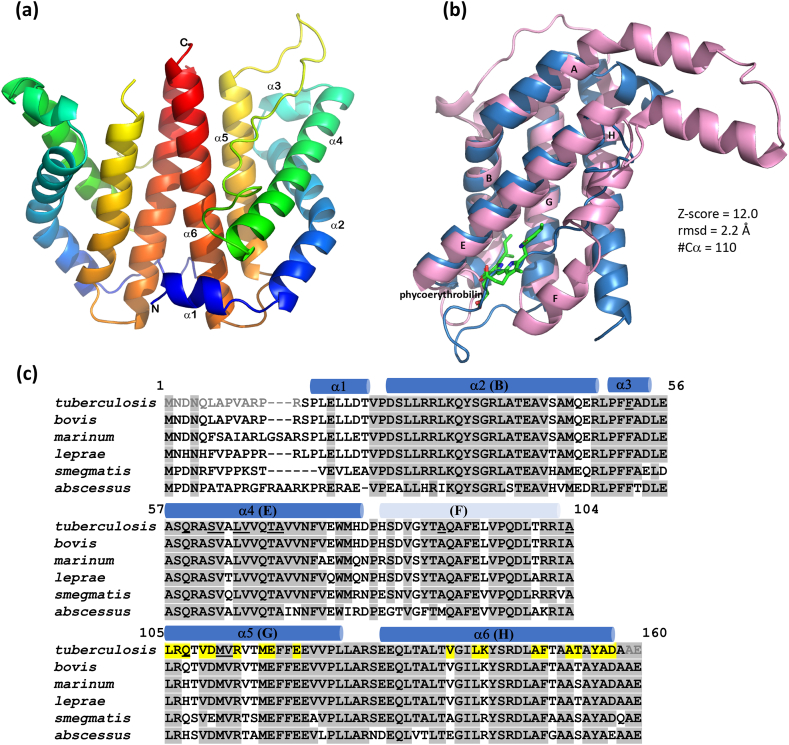
**N-MabR structure. (a)** Structure of *Mycobacterium tuberculosis* MabR. Ribbon representation of the N-MabR dimer, with the ribbon of each monomer colored from N- to C-termini in a rainbow spectrum (blue to red). α Helices, as well as the N- and C-termini, are labeled. Helix boundaries are Pro^15^-Thr^21^ (α1), Asp^24^-Arg^48^ (α2), Pro^50^-Asp^54^ (α3), Ala^57^-His^79^ (α4), Arg^105^-Val^122^ (α5) and Glu^130^-Asp^157^ (α6). **(b)** Comparison of N-MabR with a nonheme globin sensor. Superimposition of N-MabR (blue ribbon) with *Phormidium rubidium* phycoerythrin (pink ribbon; PDB ID 5nb3). The chromophore moiety PEB166α is depicted in stick representation, with carbon, oxygen, and nitrogen atoms colored green, red, and blue, respectively. Helices are labeled according to the standard globin helical nomenclature. Note that the helix F was not observed in the N-MabR structure. Information on the superimposition quality, including *Dali* Z-score, rmsd in Å, and the number of superimposed Cα atoms (#Cα), is provided. Ribbon images were produced using *PyMOL* software (Schrodinger, LLC). **(c)** Multiple sequence alignment of N-MabR orthologues. The *Mycobacterium* species included are *tuberculosis*, *bovis*, *marinum*, *leprae*, *smegmatis,* and *abscessus*. Numbering is based on the *tuberculosis* sequence. Quasi-strictly conserved residues (i.e., conserved in at least 5 out of 6 species) are shaded in gray, while residues involved in the dimerization interface in the N-MabR crystal structure are highlighted in yellow in the *tuberculosis* sequence. Residues not modeled in the crystal structure are shown in gray in the *tuberculosis* sequence. α Helices found in the N-MabR structure are indicated above the sequences as blue cylinders and labeled. The expected location of helix H, according to the canonical globin fold scheme, is marked in pale blue. In the *tuberculosis* sequence, residues interacting with the chromophore moiety PEB166α in the α-subunit A of the phycoerythrin complex structure are underlined. (For interpretation of the references to color in this figure legend, the reader is referred to the Web version of this article.)

The N-MabR structure consists of a bundle of four long α-helices (α2, α4, α5, and α6) and two shorter α-helices (α1 and α3) (Fig. 1a). The helix α2 (residues Asp^24^-Arg^48^) is kinked by approximately 60° at residue Ser^34^.

Interface analysis using the *PDBePISA* tool revealed stable homodimers formed across crystallographic symmetries, with an average solvation free energy gain of -19.2 kcal mol^−1^ upon dimer formation and a maximum Complexation Significance Score (CSS = 1) (Fig. S5). The dimeric interface is formed by the last two antiparallel helices, α5 and α6, burying a surface area of over 1000 Å^2^ (Fig. 1a). This interface is primarily stabilized by hydrophobic contacts and salt bridges, with 37 % of the interfacing residues being charged and 33 % hydrophobic (Figs. S5 and S6). Additionally, the low *PISA* ΔG p-values for the observed solvation free energy gain suggest that these interfaces are likely genuine protein-protein interactions rather than crystal packing artifacts.

### 2.2 N-MabR is a nonheme globin sensor domain

N-MabR was identified as a globin fold using the *Dali* server, an unexpected result given the lack of sequence similarity to any structure of PDB. The top match (*DALI* Z-score = 12) was the subunit β of C-phycoerythrin from the marine cyanobacterium *Phormidium rubidium* (PDB ID 5nb3) [15], with an rmsd of 2.2 Å over 110 superimposed Cα atoms (Fig. 1b). This protein belongs to the phycocyanin family, which is known to bind chromophore molecules in cyanobacteria and red algae [16]. Several other nonheme-binding phycocyanin family structures were among the top matches, followed by other nonheme globin structures, such as the *Bacillus subtilis* RsbR regulator (PDB ID 2bnl) [17], and the plasmid-encoded protein pXO1-118, known to reduce sporulation in *Bacillus anthracis* (PDB ID 3pmd) [18]. Many heme-containing globin sensors also appeared in the results.

Notably, residues crucial for metal and heme binding in globin sensors were not structurally conserved in N-MabR. Histidine residues essential for iron chelation lacked structural equivalents in MabR (Fig. S7). The absence of residues for heme/Fe chelation, along with the closest structural similarity to nonheme globin sensors, suggests that N-MabR likely functions as a nonheme globin sensor.

Finally, based on the classical globin numbering scheme [19], helices α2, α4, α5 and α6 can be labeled B, E, G and H, respectively (Fig. 1c). N-MabR adopts a “truncated’ 2/2 globin fold, a simplified version of the classical 3-on-3 α-helical sandwich, characterized by a 2-on-2 α-helical sandwich of the helix pairs B/E and G/H [20]. Helix F, typically involved in closing the heme/ligand-binding pocket, was not observed in the N-MabR crystal structure, possibly due to the structure being in an *apo* form.

### 2.3 C-MabR structure reveals two distinct domains

The crystal structure of C-MabR was solved a combination of single-wavelength anomalous diffraction (SAD) phasing and molecular replacement (MR) [21]. The structure was refined to a resolution of 2.03 Å, with final R-factor and R-free of 0.197 and 0.254, respectively. The crystallographic asymmetric unit contains a single protein molecule. The final model exhibited excellent stereochemical qualities, with no residue falling outside the allowed regions and a *MolProbity* score of 1.43, placing it in the 98th percentile compared to structures of similar resolution.

**Fig. 2:**
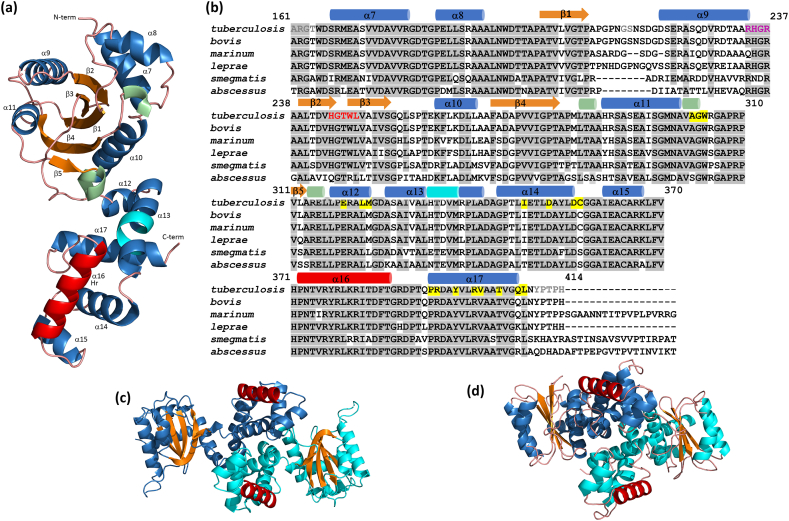
**C-MabR crystal structure. (a)** Ribbon representation of the C-MabR structure. α-helices, β-strands, 3.10 helices, and the π-helix are shown in blue, orange, light green, and cyan, respectively. The recognition helix (Hr) of the HTH motif (i.e., the second helix of the HTH motif that inserts into the major groove of the B-DNA promotor) is highlighted in red. α-helices and β-strands are labeled with the following boundaries: α7, Ser^167^-Arg^179^, α8, Pro^184^-Ala^191^, β1, Ala^201^-Pro^208^, α9, Asp^220^-Arg^234^, β2, Ala^239^-His^244^, β3, Trp^247^-Ser^253^, α10, Lys^261^-Leu^267^, β4, Phe^270^-Thr^280^, α11, His^288^-Ala^300^, β5, Val^311^-Leu^312^, α12, Leu^317^-Met^323^, α13, Ala^326^-Leu^332^ and Arg^338^-Ala^341^, α14, Gly^344^-Asp^356^, α15, Ile^361^-Lys^367^, α16, Pro^372^-Thr^386^, α17, Pro^393^-Gln^407^. **(b)** Multiple sequence alignment of C-MabR orthologues. The figure legend and coloring scheme are described in Fig. 1c. Secondary structures are shown above the sequences as cylinders (helices) and arrows (strands), colored according to panel (a). The catalytic and inhibitory sites of the GGDEF domain, identified by structural similarity, are indicated in red and magenta, respectively. **(c)** Ribbon view of the C-MabR crystal dimer. The dimer results from the arrangement of molecules in the crystal. The molecule in the asymmetric unit is shown in blue, and the one generated by symmetry is colored in cyan. Recognition helices in both monomers are shown in red, with β-strands in orange. **(d)** Ribbon view of a PucR-type regulator. The dimer shown is from the crystal structure of the polyketide synthase regulator BAD_0249 from *Bifidobacterium adolescentis* (PDB ID 3onq). The dimers in panels (c) and (d) superimpose with an rmsd of 4.9 Å over 389 superimposed Cα atoms (*Dali* Z-score of 27.8), using the *Dali* server (http://ekhidna2.biocenter.helsinki.fi/dali/). The coloring scheme is the same as in panel (c). Ribbon images were generated using *PyMOL* software (Schrödinger, LLC). (For interpretation of the references to color in this figure legend, the reader is referred to the Web version of this article.)

C-MabR consists of 11 α-helices and a β-sheet made up of 5 β-strands in a mixed arrangement of parallel and antiparallel stands, with the following topology: +2X, −1, -2X, -1X [22]. Additionally, three 3_10_ helices, each consisting of 3-residues, are present, as along with a π-helix (also known as a 5-helix) located within the helix α7, spanning His^333^ to Met^337^ (Fig. 2a). A short β-hairpin (Gly^245^-Thr^246^) with a type II’ β-turn connects strands β2 and β3. A disulfide bridge is found between Cys^357^ and Cys^364^. A loop region is highly flexible, with two consecutive residues (Gly^214^-Ser^215^) that could not be modeled due to missing electron density.

Overall, C-MabR is composed of two structural domains: a presumed regulatory or sensor α/β domain, and an all-α effector domain containing the HTH DNA-binding motif. These two domains are connected by two helices, α12 and α13 (Fig. 2a). Finally, aside from the PDB entry 3onq, which was used for MR-SAD phasing, no other PDB structure was found to share structural similarity with C-MabR in a *DALI* search.

### 2.4 The dimeric interface in the C-MabR crystal structure

An analysis of the crystallographic assembly using the *PDBePISA* server [23] reveals a dimeric interface generated by crystal symmetry around the y-axis [-x+1, y, -z-1/2] (Fig. 2c). The buried surface area of this interface is 1128 Å^2^, which constitutes only ∼10 % of the total protein surface area of each monomer– a relatively low percentage compared to other observed dimers [24]. The solvation free energy gain upon dimer formation, driven primarily by hydrophobic interactions (excluding hydrogen bonds and salt bridges) was calculated to be -13.3 kcal mol^−1^. Due to the symmetrical nature of the interface, reciprocal interactions occur between the two protomer chains (Fig. S8). A total of 29 residues contribute to the dimeric interface in C-MabR, mainly located in helices α6 and α11, as well as the C-terminal region of helix α8 (Ala^352^-Gly^358^) and the loop Ala^300^-Gly^306^.

While the *PISA* significance score is modest at 0.151, the *PISA* ΔG p-value for the observed solvation free energy gain (0.233) supports the likelihood that the identified C-MabR dimeric interface is genuine, rather than an artefact of crystal packing artifacts (Table S2). Interestingly, a similar dimeric interface is also present in the only other solved crystal structure of a PucR-like transcriptional regulators (PDB ID 3onq) (Fig. 2d). In this structure, four molecules in the crystallographic unit form two dimers, with interface properties comparable to those observed in C-MabR, though involving entirely different interfacing residues (Table S2).

### 2.5 C-MabR contains a GGDEF domain fold without catalytic and inhibitory sequence motifs

The MabR sequence is annotated in the InterPro database as containing two distinct protein signatures: a GGDEF-like domain (entry PF17853/IPRO041522) and a PucR C-terminal HTH domain (entry PF13556/IPRO025736) [25]. The GGDEF-like domain of MabR adopts the canonical fold of a diguanylate cyclase GGDEF domain, which comprises five anti-parallel β-strands (β1-β5) surrounded by five α-helices (Fig. 2a) [26]. In catalytic GGDEF domains, the substrate-binding site (A site) containing the conserved GGDEF motif is located in the β2-β3 loop, while the product-binding RXXD inhibitory site (I site) is found in the loop between β2 and the preceding helix. However, MabR's GGDEF-like domain is unlikely to possess diguanylate cyclase activity, as the corresponding regions display entirely different amino acid sequences: HGTWL for the A site and RHGR for the I site (Fig. 2b).

### 2.6 **The MabR domain architecture, combining globin, GGDEF-like, and HTH modules, is unique to the Actinomycetia class of bacteria**

The MabR structure integrates three distinct structural modules: a non-heme globin, a GGDEF-like domain, and an HTH motif. Sequence similarity searches using BlastP reveal that homologous sequences are found almost exclusively in Actinomycetes class of bacteria, which includes orders such as Mycobacteriales, Propionibacteriales, Streptomycetales, and Bifidobacteriales. Similarly, structural similarity searches using *Foldseek* indicate that proteins containing all three domains are predominantly found in Actinomycetia, while many other proteins possess one or two of these domains (Table S3). In summary, only MabR-type proteins are characterized by the unique combination of a globin fold, GGDEF-like domain, and HTH motif, and this specific protein architecture is largely restricted to Actinomycetia bacteria, with few exceptions (Table S3). Moreover, a single species can contain multiple MabR-type proteins. For example, in *Mtb*, in addition to MabR (*Rv2242*), four other regulators - *Rv1194c*, *Rv1429*, *Rv1453*, and *Rv2370c* - share both sequence similarities and the MabR domain architecture.

### 2.7 MBP-MabR exists as both a dimer and a tetramer as shown by gel filtration and SAXS experiments

**Fig. 3:**
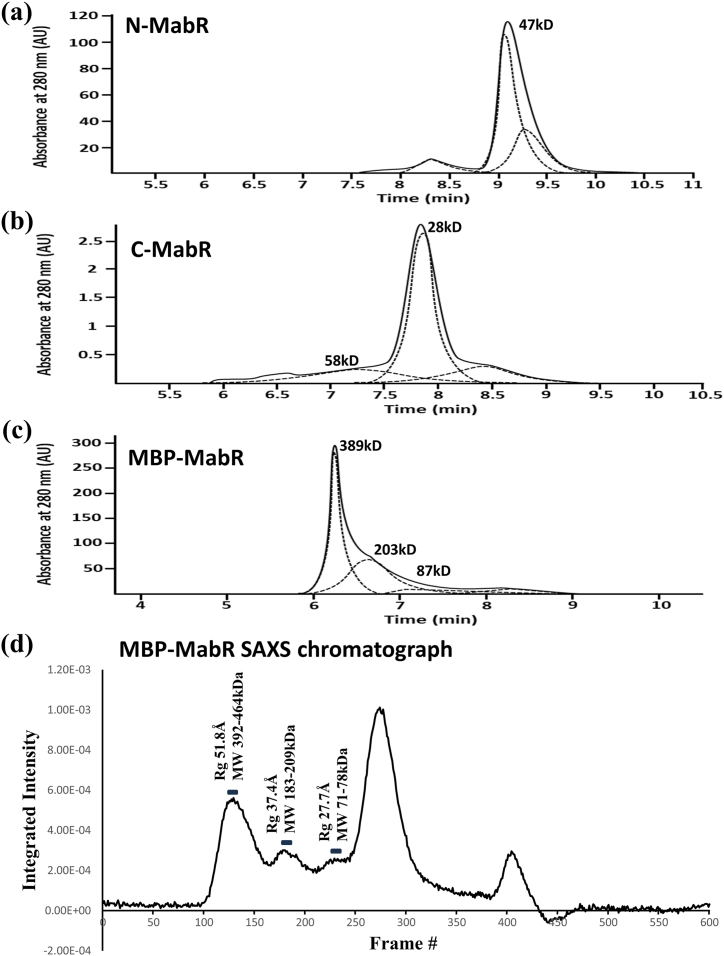
**Oligomeric states of MabR constructs.** Gel filtration chromatography elution profiles for N-MabR **(a)**, C-MabR **(b)**, and MBP-MabR **(c)**. The molecular weights of the major elution peaks are indicated, corresponding to different oligomerization states of the proteins, and estimated based on the calibration curve (Fig. S4). Peak deconvolution is shown with a dashed line.The chromatographic profiles are representative of three replicates (*n* = 3). **(d)** SEC-SAXS chromatogram of MBP-MabR, with the radius of gyration (Rg) from Guinier analysis and molecular weights estimated using four different methods shown for the three selected frame regions corresponding to the observed peaks. The large peak at frames 250–300 corresponds to the protein contaminant SlyD from *Escherichia coli* (Fig. S2), which likely did not affect the results due to its small size (20.8 kDa) compared to MBP-MabR forms. Further details on the SAXS analysis are provided in Fig. S10.

HPLC experiments reveal that C-MabR is primarily eluted as a monomer in solution, with an apparent molecular weight of 28 kDa (retention time, Rt = 7.85 min). In contrast, N-MabR elutes as a dimer with an apparent weight of 47 kDa (Fig. 3). The minor peak at 58 kDa in the C-MabR profile likely corresponds to the dimeric form, as re-injecting the monomeric peak after 24h produces the same chromatographic profile.

For MBP-MabR, gel filtration shows two main peaks with retention times of 6.24 and 6.63 min (Fig. 3). These peaks, with estimated molecular weights of 389 and 203 kDa, correspond to tetrameric and dimeric forms, respectively. A small amount of monomer is assumed to elute at a retention time of 7.15 min, giving the following relative proportions based on peak areas: 58 % tetramer, 35 % dimer, and 7 % monomer. These proportions remain constant across a tested concentration range of 0.1–5.6 μM (Fig. S9a). Additionally, re-injecting the isolated tetramer peak after 48 h results in an identical gel filtration profile, suggesting an equilibrium between the oligomeric forms. However, after 3 weeks, small changes favoring the tetramer are observed, with its relative proportion increasing from 58 % to 64 % (Fig. S9b).

Size-exclusion chromatography coupled with small-angle X-ray scattering (SEC-SAXS) further confirms that MBP-MabR exists in three distinct oligomeric forms (Fig. 3d): primarily as tetramers with an estimated radius of gyration (Rg) of 51.8 Å (Guinier fit R^2^ = 0.99), followed by dimers (Rg = 37.4 Å, Guinier fit R^2^ = 0.95), and a minor population of monomers (Rg = 27.6 Å, Guinier fit R^2^ = 0.93) (Fig. S10). Molecular weight calculations from SAXS data, using four different methods, support this distribution of oligomeric forms (Table 2).

**Table 2:** SEC-SAXS data summary for MBP-MabR tetramer, dimer, and monomer.

	tetramer	dimer	monomer
*Guinier Rg (Å)*	51.85 ± 0.55	37.36 ± 0.61	27.73 ± 0.39
*qmin∗Rg*	0.497	0.409	0.342
*qmax∗Rg*	1.324	1.312	1.328
*Guinier fit R*^*2*^	0.992	0.952	0.935
*MW Vp (kDa)*	463.9	209.1	76.0
*MW Vc (kDa)*	413.9	191.8	71.0
*MW S&S (kDa)*	408.8	182.9	73.8
*MW Bayes (kDa)*	392.3	208.0	78.5
*Theoretical Rg from AF2 model (Å)*	61.51	41.75	37.51
*χ*^*2*^*goodness-of-fit test value*	2.803	1.399	2.280

### 2.8 Predicted *AlphaFold* models of MabR dimer and tetramer forms

**Fig. 4:**
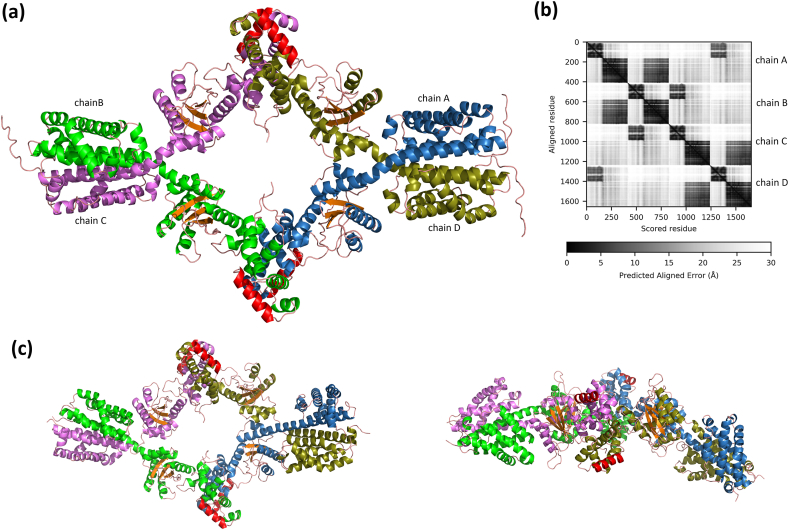
**Model of the MabR tetramer. (a)** Ribbon representation of the MabR tetramer *AF2* model. β-strands are colored orange, α-helices are colored blue, green, magenta, and dark green for chains A, B, C, and D, respectively. The recognition helix of the HTH motif is depicted in red. The model shown here is the most confident one based on predicted local distance test (pLDDT) scores among the five models generated. **(b)** Predicted aligned error plot of the MabR tetramer *AF2* model. Predicted aligned error (PAE) estimates the positional distance error in Å at residue x when the predicted and the true structures are aligned on residue y. The PAE 2D heatmap highlights regions of the predicted model for which the relative positions and orientations are confidently predicted. These regions are visible as dark blocks with PAE values below 5 Å for pairs of interacting residues. The circular interacting organization of the MabR tetramer is evident in the PAE 2D plot: the C-terminal part of chain A interacts confidently with the C-terminal domain of chain B, the N-domain of chain B interacts confidently with the N-domain of chain C, and so on. **(c)** Two views of the MabR tetramer, rotated 90° along the X-axis, obtained from MD simulations of the *AF3* model. The coloring scheme is the same as in (b). (For interpretation of the references to color in this figure legend, the reader is referred to the Web version of this article.)

The tetramer model of the FL-MabR, generated by artificial intelligence programs *AF2* and *AF3*, reveals a structural organization where each MabR molecule engages two dimeric interfaces, forming a circular assembly (Fig. 4). In this configuration, the C-domain of chain A pairs with the C-domain of chain B, the N-domain of chain B pairs with the N-domain of chain C, and the C-domain of chain C interacts with C-domain of chain D, continuing this pattern throughout the tetramer. Within this assembly, the C-domains are crossed, interlocking with the N-domain dimer interface.

the confidence score of the *AF* model is very high for the C-domains (pLDDT >90), and high for the N-domains (pLDDT >70), but low for the linker regions connecting these two domains (pLDDT <70), suggesting relative conformational flexibility between the two domains (Figs. S11 and S12). This flexibility may explain why successful crystal formation was only achieved with isolated fragments of N- and C-domains. Importantly, the two dimeric interfaces in the *AF* tetramer models closely resemble those observed in the crystal structures of the individual domains (Table S4).

When instructing *AF* to generate a dimer of FL-MabR, *AF2* produced two distinct models: one with a dimeric interface involving only the C-domain, and another with dimeric interfaces at both the two N- and C-domains, like the tetrameric models (Figs. S12 and S13). In contrast, *AF3* produced a single dimeric model, featuring an N-domain interface with a crossed organization similar to the tetrameric models (Table S4). Notably, when using the MBP-MabR construct, only the dimer utilizing both interfaces was obtained, i.e., *AF2* generating an uncrossed structure, while *AF3* produced a crossed structure.

To assess the structural stability of the *AF* models, MD simulations were performed. The simulations demonstrated that both the dimer and tetramer forms of MabR exhibit high overall structural stability, as shown in Fig. 4c. While the N- and C-domains remain individually stable, their relative positions show more variation (Table S5 and Fig. S14).

Additionally, we calculated the theoretical SAXS curves using *CRYSOL* for the MBP-MabR *AF2* models of the tetramer, dimer, and monomer. These theoretical scattering profiles were then fitted to the experimental SAXS data of corresponding peaks in the SEC-SAXS profile (Fig. 3d). The fits yield χ^2^ goodness-of-fit test values of 2.8, 1.4, and 2.3 for the tetramer, dimer, and monomer, respectively (Table 2 and Fig. S15). Although these values slightly deviate from the ideal target value of ∼1 for a statistically optimal fit in *CRYSOL*, the discrepancy likely arises from the equilibrium between the different oligomeric forms, leading to a polydisperse nature within the MBP-MabR fractions of the SEC-SAXS profile.

### 2.9 Electron microscopy analysis of MabR

**Fig. 5:**
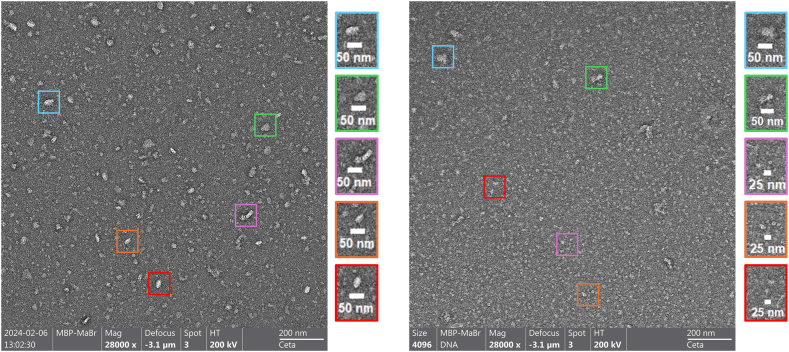
**Transmission electron microscopy imaging of MBP-MabR protein and MBP-MabR/DNA complex, visualized using negative staining.** (a) Native MBP-MabR protein at a concentration of 1.2 μM. (b) MBP-MabR/DNA complex at a 1:4 molar ratio (protein concentration 1.2 μM), examining the effect of DNA binding on the protein's oligomerization state. The analyses were performed using a Talos FEG 200 keV microscope and Ceta-D camera at 28k magnification. Images were captured with Velox software.

Electron microscopy analyses using uranyl acetate staining and transmission electron microscopy (ns-TEM) were performed on a sample of MBP-MabR to observe its oligomerization states. The analysis (Fig. 5a) revealed particles with an average size of 40–50 nm, suggesting significant oligomerization, especially considering that a monomer of MBP-MabR is estimated to have a diameter of 7–12 nm based on *AF* models. The particles were well dispersed, with minimal aggregation observed. The protein adopted various conformations, predominantly straight, consistent with the *AF* tetramer models (Fig. 4).

To investigate the effect of DNA binding, a 23-bp double-stranded DNA segment was incubated with the protein at the same concentration before negative staining. This resulted in an increased number of particles and a reduction in their size by half (Fig. 5b). Given that the stoichiometry remained unchanged, these findings suggest a disruption of the higher-order oligomeric states, leading to a dimeric form. The dimeric particles displayed different conformations, appearing more spherical or tubular. Thus, it appears that the interaction with DNA disrupts the tetrameric organization of MBP-MabR, favoring a dimeric form, which is likely the functional state for DNA binding.

### 2.10 MabR DNA-binding activity

**Fig. 6:**
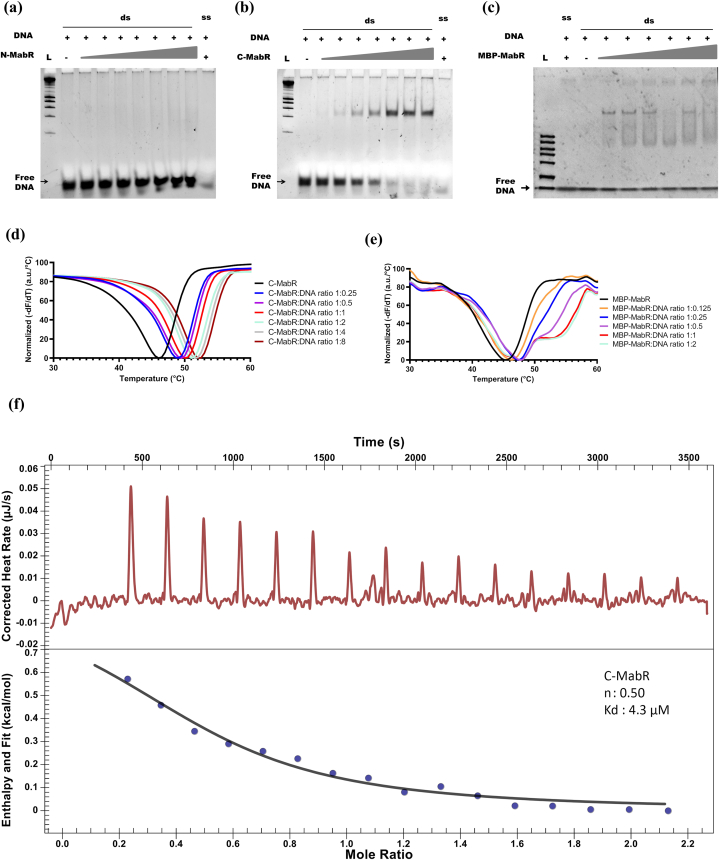
**DNA-binding activity of MabR constructs. (a**–**c)** EMSA of N-MabR, C-MabR, and MBP-MabR with 23-bp double-stranded (ds) and single-stranded (ss) DNA fragments. ‘L’ denotes the DNA ladder. (d–e) TSA of C-MabR and MBP-MabR with 23-bp ds DNA. Normalized inverse first derivative melting curves are shown, averaged from 8 replicates for C-MabR/DNA and 4 replicates for MBP-MabR/DNA complexes. **(f)** ITC analysis of C-MabR binding to DNA. The upper panel shows the baseline-corrected raw data for the interaction of C-MabR with 23-bp double-stranded DNA. The lower panel displays the integrated ITC data fitted to an independent model of binding. The estimated binding stoichiometry (molar ratio of DNA to C-MabR) and the dissociation constant (K_d_) are indicated.

To evaluate the DNA-binding capacity of MabR constructs, EMSA experiments were performed (Fig. 6). As anticipated, N-MabR, lacking the HTH DNA-binding motif, exhibited no DNA-binding activity. Conversely, C-MabR displayed clear DNA-binding activity, as evidenced by a distinct band shift, indicating the formation of a protein-DNA complex. However, EMSA analysis of MBP-MabR was less straightforward. The persistent free DNA band and the emergence of two shifted bands suggested weaker binding and potential non-specific interactions. Additionally, weak binding to single-stranded DNA further complicated interpretation.

Thermal denaturation studies were conducted to assess the stability of C-MabR and MBP-MabR in the presence of increasing concentrations of the target DNA promoter. C-MabR exhibited a concentration-dependent shift in its melting temperature (Tm), allowing the estimation of an apparent dissociation constant (K_d_) of approximately 1 μM at 46 °C (Fig. S16). However, due to the complexities observed in EMSA, the thermal denaturation curves for MBP-MabR were difficult to interpret, showing at least two distinct Tm values.

To further investigate the interaction between MabR and DNA, we performed ITC measurements. C-MabR displayed a clear binding profile, forming a 1:2 DNA-to-protein complex with an apparent K_d_ of 4.3 μM (Fig. 6f), consistent with the TSA findings (Fig. 6d). In contrast, the ITC analysis of MBP-MabR binding to DNA revealed a more complex pattern (Fig. S17), similar to the TSA data (Fig. 6e). The ITC results indicated multiple thermodynamics events, suggesting a more intricate binding mechanism (Fig. S17).

## 3 Discussion

The mycomembrane, a unique barrier composed of long-chain fatty acids (C_60_ to C_90_), the so-called MA, is essential for the virulence and persistence of mycobacterial pathogens. Disrupting its biosynthesis is a well-established strategy for developing anti-tuberculosis drugs [4]. While several agents target fatty acid biosynthesis, no drug candidates have been proposed to specifically address MA biosynthesis control, as the underlying mechanisms regulating this process remain incompletely understood.

Only three regulators have been identified as controlling MA biosynthesis: FasR, MabR, and MadR. FasR (*Rv3208*) activates the *fas*-I operon, MabR (*Rv2242*) regulates the *fas*-II *fabD*-*acpM*-*kasA*-*kasB* gene cluster [9,27], and MadR (*Rv0472c*) controls MA desaturation and biosynthesis in response to cell surface perturbation [28]. Of these, MabR remains the least characterized.

Transposon studies have shown that the *mabR/Rv2242* gene is essential for *in vitro* growth of the H37Rv strain [[29], [30], [31], [32]], but not the CDC1551/Oshkosh strain [33]. While a formal confirmation of MabR's essentiality in an *in vivo* animal model of tuberculosis is lacking, its potential as a drug target for disrupting mycomembrane production at the level of the fatty acid elongation pathway warrants further investigation.

In this study, we experimentally determined the crystal structures of MabR's N- and C-terminal domains, finding them to be highly consistent with *AF2* predictions (Cα rmsd of 0.7–1.3 Å and 1.2 Å, for N-MabR and C-MabR, respectively). These structures add to the growing body of evidence supporting AF2's accuracy [34].

The C-MabR structure adds a second crystal structure of the PucR C-terminal HTH domain family, in addition to the *Bifidobacterium adolescentis* BAD_0249 structure (PDB ID 3onq). N-MabR adopts a globin fold, while C-MabR features a GGDEF-like domain and an HTH DNA-binding domain. The MabR's unique domain combination is not found in other regulators and is exclusive to Actinomycetia bacteria. This suggests a potentially novel regulatory paradigm. Like many bacterial regulators, MabR's DNA-binding activity is likely modulated by effector molecules. Both the nonheme sensing globin domain and GGDEF-like domain could serve as potential binding sites for such effectors, which remain to be identified.

While we were unable to solve the full-length MabR structure, MBP fusion construct revealed that MabR predominantly forms tetramers and dimers in solution. Crystal packing analysis identified dimeric interfaces specific to each MabR domain. Interestingly, the dimeric interface observed in the C-terminal domain is similar to that found in the only previously deposited crystal structure of a PucR protein. Moreover, *AF2* and *AF3* independently predicted dimeric interfaces like those observed in our crystal structures, further validating our findings (Table S4).

The influence of MabR's diverse oligomeric states on DNA binding remains unclear. While electron microscopy suggests that MabR favors a dimeric form for DNA interaction, this observation lacks corroboration from electrophoresis gel analysis. The ITC data for MBP-MabR binding to DNA revealed multiple thermodynamic events (Fig. S17), likely reflecting the interplay between DNA binding and the tetramer-to-dimer dissociation of MBP-MabR observed by NS-TEM (Fig. 5). Together, the ns-TEM, TSA, and ITC findings support a model in which MabR preferentially binds DNA in its dimeric form.

It should be noted that both dimeric and tetrameric forms of MabR are compatible with its binding to a palindromic DNA sequence. The distance between the recognition helices of the two HTH motifs in different monomers is most compatible with binding to two adjacent major grooves of B-DNA (i.e., 34 Å, corresponding to one complete turn) (Table S6). This compatibility is observed in the crystal structure of C-MabR and in the *AF2*/*AF3* structural models, except for the *AF3* model of the MabR dimer, which involves a crossed organization through the N-domain dimeric interface (Fig. S12). Therefore, the relationship between MabR's oligomerization states and its DNA binding mode remains unresolved.

The tetrameric form of MabR observed in *in vitro* assays might be an artefact of the high protein concentrations used, which may exceed physiological levels. However, data from the PaxDb protein abundance database (version 5.0) [35] suggests that the relative abundance of MabR in cells ranges from 23 to 59 ppm. Converting this to molar concentration using the established formula [36], we find that MabR concentration in cells are estimated to be between 0.115 and 0.295 μM. This range is consistent with the concentrations used in our SEC experiments, suggesting that the tetrameric form of MabR may indeed exist *in vivo*.

This study has certain limitations. Our inability to obtain the crystal structure of full-length MabR necessitated the use of an MBP-fused variant to study its oligomeric states. While MBP is generally not known to form oligomers, its presence could potentially influence the equilibrium between dimer and tetramer of MabR.

We lack direct experimental evidence for the existence of the two dimeric interfaces within the tetramer form of MabR. Our findings rely on gel filtration, SAXS analysis and predictive modeling using *AF2* and *AF3*. Although *AF* models may have low confidence scores in linker regions, indicating potential inaccuracies in the generated structural organizations. Specifically, the accuracy metrics pTM and ipTM from *AF3* suggest likely failed oligomerization predictions (Fig. S12). Despite this, the two dimer interfaces on which these models are based remain reliable. The difficulty of *AF* programs in generating a single, stable structural model is likely due to the conformational flexibility between domains, leading to multiple possible oligomeric organizations. Future cryo-EM and molecular dynamics studies are expected to validate the predictive modeling of MabR oligomerization.

Our findings prompt several intriguing questions. Determining the dimeric interface most relevant to MabR's DNA-binding activity is a priority. Resolving the structure of full-length MabR in complex with DNA via cryoEM single-particle analysis will be essential to clarify the link between MabR's oligomerization states and its DNA-binding mode. Furthermore, identifying small molecules that bind to MabR and modulate its DNA-binding activity presents an exciting avenue for future research. These questions are central to our ongoing investigations.

In conclusion, MabR's critical role in bacterial growth makes it a promising target for drug development. The structural information provided in this study, including the crystal structure of MabR and its oligomeric states in solution, offers a valuable foundation for future drug design efforts aimed at disrupting mycomembrane biosynthesis and combating mycobacterial infections.

## 4 Materials and methods

### 4.1 Protein production and purification

Initially, we attempted to work with the full-length MabR protein. However, due to its poor stability during purifications, we decided to explore other constructs. Using four different domain prediction algorithms, we identified a consensus boundary around position 160 (Table S1), which led us to include both N- and C-terminal fragments in our study.

Synthetic genes of full-length MabR (FL-MabR), as well as the N-terminal (residues 1–160; N-MabR) and the C-terminal (residues 161–414; C-MabR) domains, were synthesized and cloned *de novo* into the bacterial expression plasmid pET28a at the *Nde*I and *Eco*RI cloning sites, resulting in N-terminal 6x His-tagged recombinant proteins (GeneCust, Boynes, France).

Additionally, we included a final construct consisting of an N-terminal *E. coli* maltose-binding protein (MBP) fused to the full-length MabR (MBP-MabR), cloned into the pET15b plasmid, which produced a soluble protein. The MBP sequence was modified to enhance protein stability and crystallization [37]. The MBP tag was kept as its removal led to the precipitation of MabR. Additionally, we retained the MBP tag in an attempt to use it as a crystallization chaperon [37], although this approach was ultimately unsuccessful. Details on the four constructs are provided in Supplementary Fig. S1.

*E. coli* BL21(DE3) cells transformed with the respective plasmids were grown in Luria-Bertani (LB) medium at 37 °C with 50 μg⁄mL kanamycin or ampicillin. When the culture reached an optical density of 0.8 at 600 nm, protein expression was induced by adding 1 mM IPTG, followed by overnight growth at 28 °C. The cells were then harvested by centrifugation at 4000 rpm for 20 min, and the pellets were stored at -20 °C before purification.

The cell pellets were resuspended in a lysis buffer containing 0.6 M NaCl, 20 mM HEPES pH 7.5, 10 % glycerol, 5 mM β-mercaptoethanol, 10 mM imidazole, and a Roche cOmplete™ EDTA-free protease inhibitor tablet. Cells were lysed on ice via sonication using a Sonics VCX-500 machine for 40 cycles, each consisting of a 10-s pulse at 30 % amplitude followed by a 20-s rest period. The lysate was then centrifuged at 28,000 rpm for 45 min and the supernatant was incubated for 2 h at 4 °C with cobalt chelating resin (Talon® Superflow, GE Healthcare), pre-equilibrated with lysis buffer.

The resin was collected in a PD10 column and washed in two steps: first with a buffer containing 1 M NaCl, 20 mM HEPES pH 7.5, 10 % glycerol, 5 mM β-mercaptoethanol, and 30 mM imidazole, followed by a second wash with an increased imidazole concentration of 40 mM. Protein was eluted using with a buffer containing 0.4 M NaCl, 20 mM HEPES pH 7.5, 5 % glycerol, 5 mM β-mercaptoethanol, and 400 mM imidazole.

Protein purity was assessed by 4–12 % SDS-PAGE, and protein identification was confirmed by mass spectrometry was used after tryptic digestion of in-gel proteins. A major contaminant, identified by mass spectrometry analysis as the *E. coli* cis-trans isomerase SlyD (Fig. S2), was typically removed by adding a gel filtration chromatography step to the purification process. Protein concentrations were measured using a NanoDrop One spectrophotometer (ThermoFisher) with extinction coefficients calculated from the *ProtParam* server [38].

### 4.2 Protein crystallization and diffraction data

N-MabR sample, concentrated at 17 mg/ml, was screened for crystallization conditions by vapor diffusion method at 20 °C in hanging drops of mixing 1:1 μL of crystallization: protein solutions with several commercial kits from Hampton Research (CA, USA) and Molecular Dimensions (Sheffield, UK). Initial crystals of poor diffraction quality (i.e., ∼10.0–6.8 Å resolution) were obtained in 200 mM NaBr 100 mM Bis-Tris propane pH 8.5 and 20 % (w/v) PEG3350. After optimization, the best crystals were grown by replacing NaBr salt with Na-malonate.

During the C-MabR purification, we noticed that a high protein sample concentration tended to form crystals. These crystals were fortunately found to diffract X-rays. Therefore, suitable crystals of C-MabR were produced by standing overnight at 4 °C, and the protein solution (2 mg/ml) was eluted at 400 mM imidazole from cobalt beads. Considering how fast the crystals were produced, we observed that the most diffracting ones were the smallest. Crystals of N- and C-MabR are shown in Fig. S1.

Crystals were flash cooled with liquid nitrogen in the corresponding crystallization buffers supplemented with 25 % glycerol. All diffraction experiments were conducted at the SOLEIL synchrotron facility (Paris, France). Diffraction data of C-MabR crystals were indexed with *XDS* [39] using the automated data-processing *XDSME* [40]. For N-MabR, collected data were analyzed with *autoPROC* [41], and due to significant anisotropy in diffraction limits, i.e., 3.0 Å in the c∗ axis and 3.9 Å in the a∗ and b∗ axes, Bayesian estimation of structure amplitudes and anisotropic correction were applied using *STARANISO* [12].

All crystallographic screens with FL-MabR or MBR-MabR fusion protein remained unsuccessful.

### 4.3 Crystal structure determination

Molecular replacement (MR) method was used to solve the N-MabR crystal structure using as a template a predicted model of Rv2242 protein (fragment Leu^18^-Tyr^155^) taken from *AlphaFold* Protein Structure Database (https://alphafold.ebi.ac.uk; assessed in September 2021) [34]. A refinement protocol adapted to low-resolution data was used. The R_free_ reflections were randomly selected before structure determination at the early indexation stage (number of R_free_ reflections = 535, counting for 4.9 % of total reflections). MR was performed with *Phaser-2.8.3* [42], and initial refinement was started with 200 steps of jelly-body restraints via the LOw REsolution Structure Refinement pipeline (*LORESTR*) [43], followed by several tens of cycles of model building in *Coot* [44] and jelly-body refinement through *LORESTR*/*REFMAC5* [45].

MR attempts, utilizing either the PDB ID 3onq (sequence identity of 30 % with MabR) or the *AF2* model of MabR fragment Ala^161^-His^414^, proved unsuccessful in resolving the phase problem. Useful isomorphous mercury derivatives were prepared by soaking crystals in buffer solution supplemented with 10 mM HgCl_2_ for 10 min at room temperature. The mercury-derivative crystal diffracted up to 2.45 Å resolution. However, the theoretical anomalous signal was relatively weak. Several Hg sites were found with *SHELXD* [46]*,* but only at low occupancies and with the best anomalous site positioned at a 2-fold axis, strongly limiting their phasing power. Fortunately, we successfully applied an MRSAD strategy of using an MR search to place a template structure's DNA-binding domain onto the electron density map derived from an initial Hg-SAD phasing. The MR search model was the C-terminal part of the regulator of polyketide synthase expression BAD_0249 from *Bifidobacterium adolescentis* (PDB ID 3onq, PDB deposition on 2010-08-30 by Kim Y, Wu R, Tan K, Morales J, Bearden J, Joachimiak A, Midwest Center for Structural Genomics, MCSG, unpublished work).

Briefly, using four Hg peaks, initial SAD phases were generated with *Phaser-2.8.3* [42], but the resulting solvent-flattened electron density map was not interpretable for model building. Phases were further improved using *Molrep* [47] to fit the C-terminal domain of the template 3onq into the SAD map. *Phaser-EP* was then used to combine SAD and MR information, performing successive cycles of substructure completion, phasing, and density modification with *Parrot* [48]. The program *Buccaneer* was finally used for automated model building [49]. Native data was further collected at 2.03 Å resolution, allowing the determination of a higher resolution structure. The native structure was then solved by molecular replacement using the Hg-derivative structure as a template and thoroughly refined by iteratively cycling through *Coot* [44] and *REFMAC5* [45]. Crystal structure refinement quality was assessed with *MolProbity* program [50]. Images of the phased electron density maps for N-MabR and C-MabR crystal structures are illustrated in Fig. S3.

### 4.4 Analytical gel filtration analysis

Protein sample homogeneity, as well as molecular weight of constructs in solution, were evaluated by size exclusion high-performance liquid chromatography (HPLC) on an XBridge Protein BEH SEC column (200 Å, 3.5 μm, 7.8 mm × 300 mm) (Waters, Belgium) for N-MabR and Protein-Pak 125 column (125 Å, 10 μm, 7.8 mm × 300 mm) (Waters, Belgium) for C-MabR and MBP-MabR, both mounted on an Agilent HPLC 1100 DAD machine. Experiments were run at room temperature, at a flow rate of 1 ml/min, with phosphate buffer (20 mM Na_2_HPO_4_, 130 mM NaCl, pH 7.0) as the running solution and monitored by absorbance at 280 nm. Calibration curves were established using the gel filtration standards from Bio-Rad (provided as Fig. S4). Peak deconvolution was obtained using the program *fityk* 1.3.1 (https://github.com/wojdyr/fityk) [51].

### 4.5 Transmission electron microscopy

Negative staining Transmission Electron Microscopy (ns-TEM) grids were prepared for oligomeric state investigation. A 3.0 μL drop of MBP-MabR protein (0.1 mg/ml) or MBP-MabR/DNA complex (0.1 mg/ml) solution was placed on a 25 s glow-discharged thin carbon-coated 200-mesh copper grid (Electron Microscopy Sciences, CF200-CU C). After 1 min, the excess solution was removed by blotting with Whattman 40 filter paper. The grid was washed by successive soaking in three drops of 15 μL distilled water on Parafilm with blotting on filter paper between each wash to remove the excess of liquid. After washing, two successive drops (15 μL/drop) of 2 % (w/v) uranyl acetate (UA) solution were applied to parafilm, and the excess solution was removed by blotting similarly. The grid remained in contact with the last UA drop for 1 min side up before the excess was removed, and the sample was air-dried at room temperature. ns-EM grids were analyzed with a ThermoFisher Talos FEG 200 keV microscope equipped with a Ceta-D camera. The micrographs were acquired using Velox software (ThermoFisher Scientific) at a magnification of 28,000X, with each pixel of the micrographs corresponding to 0.14 nm.

### 4.6 Thermal shift assay

The stability, folding, and DNA-binding of the protein samples were assessed using thermal shift assay (TSA) conducted in an iCycler RT-PCR machine (Bio-Rad, Hercules, CA, USA). SYPRO® Orange (ThermoFisher Scientific) was utilized as an extrinsic fluorescent dye for monitoring protein unfolding, at a final concentration of 5X. The excitation and detection wavelengths were set at 450–490 and 560–580 nm, respectively. Protein samples were used at a final concentration of 4.6 μM for C-MabR and 10 μM for MBP-MabR. The samples were heated from 20 °C to 80 °C at a rate of ∼2.4 °C/min. TSA experiments were performed with four replicates for MBP-MabR and eight replicates for C-MabR. The melting curves for C-MabR in presence of DNA were analyzed using the isothermal approach via the FoldAffinity web server [52].

### 4.7 Sequence and structure analysis

Four different algorithms were used to predict the structural domain in the MabR sequence (see Table S1). Instability indices (II) were calculated from the *Protparam* tool [53], considering a default stable/unstable cutoff at II = 40.00. Ortholog search and sequence alignment were performed with *BLAST* [54] and *ClustalW2* [55], respectively. Secondary structure elements were assigned with the *DSSP* algorithm [56], and cavity identification was computed through the *CASTp3.0* server [57]. Crystallographic interface analysis was performed with the *PDBePISA* tool [58], and structure comparisons were conducted using the *DALI* web server [59]. *LigPlot* ^*+*^ produced residue-residue interactions across dimeric interfaces [60]. The *FoldSeek* tool was used for parsing structurally the *AlphaFold* model database [61]. Pictures were generated using *PyMol* Molecular Graphics System (Version 2.4.0, Schrödinger, LLC).

Dimeric and tetrameric forms of the FL-MabR, with and without the MBP fusion protein, were generated using a locally implemented version of *AlphaFold2* (*AF2* version v2.3.1 downloaded from https://github.com/deepmind/alphafold) [34], used in multimer mode with sequence databases (release of February 11, 2023) but without any 3D template information from the PDB. The program was running under the miniconda virtual environment thanks to the GitHub resource https://github.com/kalininalab/alphafold_non_docker. Furthermore, the recently released *AlphaFold3* (*AF3*), which includes significant computational improvements over the previous version *AF2* [62], was also used to generate the dimeric and tetrameric forms of MabR. This was accomplished using the beta server at https://golgi.sandbox.google.com. The *AF2* and *AF3* structural models are available upon request from the corresponding author.

### 4.8 DNA-binding assays

Electrophoresis mobility shift assays (EMSAs) were carried out with synthetic 23-base oligonucleotides provided by Sigma-Aldrich (Belgium) with the following forward sequence: 5′-GTTTTGTTGGTTATATACAAAAA-3’. A DNA footprinting analysis has previously determined this sequence as containing the putative FAS-II promoter region [9]. After annealing of the two understudied complementary oligonucleotides with a thermocycler (Biometra T3000 Thermocycler, Westburg; heat to 95 °C and remain at this temperature for 2 min; then ramp cool to 25 °C over a period of 45 min), the formation of the protein-DNA complexes was monitored by incubating at room temperature for 30 min 100 ng of double-stranded DNA (final concentration of 284 nM) with protein sample at different concentrations (final concentrations in the range 0.6–20 μM) in 25 μL of binding buffer (50 mM Tris-HCl pH 7.5, 50 mM NaCl, 200 mM KCl, 5 mM MgCl2, 5 mM EDTA, 5 mM DTT) and then carrying out native 6 % polyacrylamide gels. Gels were run on ice in a vertical electrophoresis apparatus (Bio-Rad Mini-PROTEAN Tetra Cell) at 120 V for 45 min. Gels were stained with the nucleic acid stain ethidium bromide.

### 4.9 SEC-SAXS data collection and analysis

Experiments coupling size-exclusion chromatography with small-angle X-ray scattering (SEC-SAXS) were carried out on the SWING beamline at SOLEIL synchrotron, performed at an energy of 12 keV and with a sample-to-detector distance of 2000 nm. A volume de 45 μl of MBP-MabR sample (80 μM) was injected onto a Bio SEC-3-300 column pre-equilibrated with buffer Tris 50 mM pH 7.5 NaCl 150 mM and mounted on an Agilent HPLC system. The flow rate was of 0.3 ml/min and the temperature of the experiment was of 20 °C. The scattering signal of the buffer was collected in 181 frames while 601 scattering frames were collected during the elution of the sample. The individual 1D scattering curves were obtained by radially averaging the frames and subtracting the averaged buffer curve from the averaged sample scattering curve using the image analysis software Foxtrot (courtesy of SWING beamline).

The SAXS curves were further processed and analyzed using *BioXTAS RAW* 2.2.2 software [63]. The radius of gyration Rg were estimated from the Guinier plot whereas molecular weights were evaluated from four methods, namely the adjusted Porod volume (Vp) [64], the volume of correlation (Vc) [65], shape and size classification by machine learning (S&S) [66], and a Bayesian estimation based on concentration independent methods (Bayes) [67]. Note that, although SEC-SAXS peaks are not monodisperse and consist of components close in size, the deconvolution method was not applied, as these peaks are sufficient to clearly identify the three oligomeric forms of MBP-MabR. The fit between *AF2* models and experimental SAXS data was assessed by determining the discrepancy (χ^2^) between the calculated SAXS curve from a *AF2* model and the observed SAXS data using *CRYSOL* using 100 spherical harmonics and the default settings for the other parameters [68].

### 4.10 Molecular dynamics simulation

Molecular dynamics (MD) simulations of MabR were conducted using *AF* structures. Since the *AF2* models had already undergone energy minimization through MD, we focused in our analysis on the *AF3* models. To optimize simulation time, the first 10 amino acids of each monomer were removed from the *AF3* models. Protein topology files were generated using GROMACS pdb2gmx tool, which was also used to add hydrogen atoms to the models. No positional restraints were applied to the protein atoms during the simulations. The MD simulations were performed with GROMACS 2024 [69] using the CHARMM36 force field [70]. Solvation was carried out explicitly in a dodecahedral box using all-atom TIP3P water molecules. The system's total charge was neutralized with 0.15 M of NaCl to maintain physiological ion concentrations.

The methodology followed was based on previous work by our group [71]. MD trajectories were generated under periodic boundary conditions with particle mesh Ewald electrostatics, and a 1.2 nm cutoff was applied for both Coulombic and van der Waals interactions. Temperature and pressure control were managed using the Parrinello-Rahman and V-Rescale algorithms, respectively. System minimization was first performed using the steepest descent algorithm with a maximum of 500 steps and an initial step size of 0.05 nm. During equilibration, the temperature was gradually increased from 50 K to 310 K via short MD runs (10 ps at 50 K, 20 ps at 150 K, and 20 ps at 310 K), followed by a 50 ps run at 310 K and 1 bar for system relaxation. The equilibration was extended to 60 ns with a 2-fs time step at 310 K and 1 bar. For the dimer, the production run lasted 100 ns with a 2-fs time step, while the tetramer was simulated for 20 ns due to its larger size. Energy profiles and rmsd were used to monitor system evolution during both equilibration and production phases.

### 4.11 Isothermal titration calorimetry assay

To further investigate the interaction between MabR and DNA, isothermal titration calorimetry (ITC) experiments were carried out using an Affinity ITC (TA Instruments). Prior to measurement, C-MabR, MBP-MabR proteins, and the 23-bp double-stranded DNA were dialyzed into Tris-NaCl buffer (Tris 50 mM pH7.5, 150 mM NaCl). Titrations were conducted at 20 °C. In each experiment, 2 μL of DNA titrant was injected into the 200 μL protein sample cell, with stirring maintained at 75 rpm. The nominal protein concentrations in the cell were 20 μM for C-MabR and 25 μM for MBP-MabR, while the DNA concentrations in the syringe was 200 μM for C-MabR and 162 μM for MBP-MabR. Actual sample concentrations were measured post-dialysis by absorbance at 280 nm. Data analysis was performed using the NanoAnalyze software.

### 4.12 Declaration of generative AI ad AI-assisted technologies in the writing process

During the manuscript review process, the authors used ChatGPT and Gemini services to enhance English language and sentence structure without altering the content. After using these services, the authors reviewed and edited the content as needed and take full responsibility for the final content of the publication.

## CRediT authorship contribution statement

**Véronique Megalizzi:** Writing – review & editing, Visualization, Methodology, Investigation, Formal analysis, Data curation. **Abdalkarim Tanina:** Investigation. **Camille Grosse:** Writing – review & editing, Visualization, Methodology, Investigation, Formal analysis, Data curation. **Manon Mirgaux:** Visualization, Investigation. **Pierre Legrand:** Investigation. **Gaëtan Dias Mirandela:** Investigation. **Alexandre Wohlkönig:** Writing – review & editing, Conceptualization. **Pablo Bifani:** Writing – review & editing, Funding acquisition, Conceptualization. **René Wintjens:** Writing – review & editing, Writing – original draft, Visualization, Methodology, Investigation, Funding acquisition, Formal analysis, Data curation, Conceptualization.

## Resource availability

### Lead contact

Further information and requests for resources and reagents should be directed to and will be fulfilled by René Wintjens (rene.wintjens@ulb.be).

## Materials availability

All expression plasmids used in this study will be made available on request. This study did not generate new unique reagents.

## Data and code availability

• The electron density maps and atomic models of N-MabR and C-MabR have been deposited in the Protein Data Bank with the following PDB accession codes, 9FB1 and 9F80, respectively.
• The SAXS data were deposited to SASBDB with accession codes SASDU88, SASDU98 and SASDUA8, for tetramer, dimer, and monomer peaks, respectively.
• *AF2*/*AF3* models are available from the lead contact upon request.
• The paper does not report code.
• Any additional information required to reanalyze the data reported in this paper is available from the lead contact upon request.

## Declaration of competing interest

The authors declare the following financial interests/personal relationships which may be considered as potential competing interests: Rene Wintjens reports financial support was provided by Belgian Funds for Scientific Research. If there are other authors, they declare that they have no known competing financial interests or personal relationships that could have appeared to influence the work reported in this paper.
