# Supplementary material for: Domain architecture of the Mycobacterium tuberculosis MabR (Rv2242), a member of the PucR transcription factor family
Source: Heliyon. 2024 Nov 16;10(22):e40494. doi: 10.1016/j.heliyon.2024.e40494 (PMC11617747; doi:10.1016/j.heliyon.2024.e40494)

Table S1. MabR domain boundary predictions

Web server program	Domain boundaries
DoBo[a]	164, 165, 166, 341, 173
GlobPlot[b]	4-201, 224-414
Scooby[c]	124, 161, 226, 317
ThreaDomEx[d]	1-159, 160-316, 317-414

[a] DoBo, http://sysbio.rnet.missouri.edu/multicom_toolbox/Dobo.html, Eickholt et al. (2011) DoBo : protein domain boundary prediction by integrating evolutionary signals and machine learning. BMC Bioinformatics 12, 43.

[b] GlobPlot, http://globplot.embl.de, Linding et al. (2003) GlobPlot: exploring protein sequences for globularity and disorder. Nucleic Acids Res 31, 3701-8.

[c] Scooby, https://www.ibi.vu.nl/programs/scoobywww/ , Pang et al. (2008) Identifying foldable regions in protein sequence from the hydrophobic signal. Nucleic Acids Res 36, 578-88.

[d] ThreaDomEx, https://zhanggroup.org/ThreaDomEx/, Wang et al (2017) ThreaDomEx: a unified platform for predicting continuous and discontinuous protein domains by multi-threading and segment assembly. Nucleic Acids Res 45, W400-7.

Table S2. Summary of PISA interface analysis for the crystal structures of C-MabR and 3onq[a]

Protein	C-MabR	3onq
Symmetry operation	-x, y, -z-1/2	x, y, z
Number of residues at interface	29 (11.9%)	28 (11.1%)
Solvent-accessible area at interface (Å^2)	1128 (9.6%)	1115 (8.2%)
Solvation energy gain on complex formation (kcal/mol)	-13.3 (p-value=0.233)	-18.3 (p-value=0.018)
Number of hydrogen bonds at interface	6	3
Number of salt bridges at interface	2	1
Key interfacing residues	A300, G303, W304, R305, E319, L322, D352, D356, P393, Y397, R400, V401, T404, V405, L408	A146, P148, A149, E165, A167, L168, R175, Y245, T249, A252, M256
Most probable assembly	2-mer	4-mer
Complex Formation Significance Score	0.151	0.125

[a] The interface properties are averaged over the two dimers observed in the crystal structure. The percentages shown in parentheses are percent to the total number of residues and to the total surface area. The solvation energy gain at complexation is the change of the solvation energy of the structure due to the interface formation. The P-value of the solvation free energy gain (ΔG p-value) is given in parentheses. This p-value is a measure of interface specificity, showing how surprising, in energy terms, the interface is. A value of p-value>0.5 means that the interface is likely to be an artefact of crystal packing, while p-value<0.5 suggests that the interface can be interaction specific. The lower the p-value, the more likely the interface is, with le limiting case of p-value=0 means that such interface is a truly unique spot on the protein surface. Key interfacing residues are identified as those with over 50% of their buried area engaged in the protein-protein dimeric interface. Most probable assembly is the most probable multimeric state of the crystal structure according PISA analysis, whereas the complex formation significant score indicates how significant for the assembly formation the interface is. The analysis is performed with the *PDBePISA* tool (https://www.ebi.ac.uk/msd-srv/prot_int/cgi-bin/piserver).

Table S3. Foldseek result summary using the AF2 MabR model

**64.1%** of Actinomycetes class of bacteria	**65.4%** containing the complete domain architecture of MabR (i.e., the three domains Globin+GGDEFlike+HTH)
	**34.6%** without the complete MabR domain architecture
**35.1%** of other classes of bacteria	**96.4%** without the complete MabR domain architecture
	**3.6%** containing the complete MabR domain architecture

The Foldseek query was performed onto the AFDB50 database and statistics were presented over the 1000 hits obtained. The full result data is available from the lead contact author upon request.

Table S4. Comparison between dimeric interfaces of crystal and AlphaFold structures [a]

Structural model	Number of residues at interface	Solvent-accessible area of interface (Å²)	Solvation energy gain on complex formation (kcal/mol)	Hydrogen bonds at interface	Salt bridges at interface	Key interfacing residues
RX N-MabR	30 (24.3%)	1160 (7.7%)	-19.2 (P = 0.140)	R106↔A156	R106↔D157, E120↔K142, E120↔R145	R106, V109, R113, M116, E120, T134, T137, V138, L141, S144, R145, A148, F149, T153, A156
RX C-MabR	29 (11.9%)	1128 (9.6%)	-13.3 (P = 0.233)	G303↔T404, W304↔D356, L322↔N409	R305↔D352	A300, G303, W304, R305, E319, L322, D352, D356, P393, Y397, R400, V401, T404, V405, L408
AF2 tetramer N-term interface	46 (11.2%)	1738 (7.3%)	-23.2 (P = 0.100)	R113↔T150	R106↔D157, E120↔K142, E120↔R145	P15, L16, L105, V109, R113, M116, E120, L133, T134, T137, V138, L141, S144, R145, A148, F149, A152, T153, Y155, A156
AF2 tetramer C-term interface	29 (7.0%)	1046 (4.4%)	-15.5 (P = 0.117)	R305↔Q407, L322↔Y410, Y397↔T404	R305↔D352	G303, R305, L322, D352, P393, Y397, R400, V401, T404, Q407, L408
AF3 tetramer N-term interface	65 (15.5%)	2368 (10.0%)	-25.9 (P = 0.162)	L105↔D160	R101↔D230, R106↔D157, E120↔K142, E120↔R145, R162↔D166	P15, L16, L105, V109, M116, E120, L133, T134, T137, V138, L141, S144, R145, A148, F149, A142, T153, A156, A159, E160, R162, R168, M169, R189
AF3 tetramer C-term interface	32 (7.7%)	1139 4.9%)	-14.5 (P = 0.161)	G303↔T404, L322↔Y410, Y397↔T404	R305↔D352	A300, G303, G319, A321, L322, M323, D352, P393, Y397, R400, V401, T404, V405, Q407, L408
AF2 dimer #1	31 (7.5%)	1087 (5.0%)	-16.5 (P = 0.084)	L322↔Y410, Y397↔T404	R305↔D352	A300, G303, E319, A321, L322, M323, D352, P393, Y397, R400, V401, T404, V405, Q407, L408
AF3 dimer	74 (17.9%)	2666 (11.6%)	-24.7 (P = 0.250)	Q97↔D230	D98↔R223, R101↔D227, R106↔D157, E120↔K142, E120↔R145, E185↔R189	P15, R101, L105, R106, V109, M116, E120, L133, T134, T137, V138, L141, S144, R145, A148, F149, A152, T153, Y155, A156, D157, A159, R162, W165, M169, E185, R189, A192, G219, R223

**AF2 dimer #2**	**93 (20.5%)**	**3003 (14.2%)**	**-39.3 (P = 0.059)**	**E56→H333, R305↔Q407, L322↔Y410, Y397→T404**	**D54→R289, E120→K142 E120↔R145, R305↔D352**	**P12, P15, L16, E120, P124, L133, T134, T137, V138, L141, S144, R145, F149, A300, G303, E319, A321, L322, D352, P393, Y397, R400, V401, T404, V405, L408**

[a] The interface properties are averaged over the dimers observed in the considered structural model. The sign '↔' represents a symmetrical hydrogen bond or salt bridge at protein-protein interface, i.e., for instance in the C-MabR crystal structure, R305 of chain A makes salt bridge with D352 of chain B and symmetrically D352 of chain B is in salt bridge with R305 of chain A. The percentages shown in parentheses are percent to the total number of residues and to the total surface area. The solvation energy gain at complexation is the change of the solvation energy of the structure due to the interface formation. The P-value of the solvation energy gain is given in parentheses. This P-value is a measure of interface specificity, showing how surprising, in energy terms, the interface is. A value of P-value>0.5 means that the interface is likely to be an artefact of crystal packing, while P-value<0.5 suggests that the interface can be interaction-specific. The lower the P-value, the more likely the interface is, with le limiting case of P-value=0 means that such interface is a truly unique spot on the protein surface. Key interfacing residues are identified as those with over 50% of their buried area engaged in the protein-protein dimeric interface. The analysis is performed using the *PDBePISA* tool (https://www.ebi.ac.uk/msd-srv/prot_int/cgi-bin/piserver)

Table S5. Structural comparisons of MabR *AF* models following MD simulations

rmsd (Å)	N-domain[a]	C-domain[b]	Global
Tetramer AF2 vs AF3[c]	1.75	0.53	4.79
Tetramer AF2 vs AF3-MD[d]	1.60	1.25	8.46
Tetramer AF3 vs AF3-MD[e]	2.11	1.31	8.97
Dimer AF3 vs AF3-MD[f]	1.89	1.71	5.68

[a] Superimposition rmsd of Cα atoms for residue range 15-126
[b] Superimposition rmsd of Cα atoms for residue range 201-408
[c] Structural comparison of tetrameric models: *AF2* vs *AF3* models
[d] Structural comparison of tetrameric models: *AF2* vs *AF3* post-MD simulation
[e] Structural comparison of tetrameric models: *AF3* vs *AF3* post-MD simulation
[f] Structural comparison of dimeric models: *AF3* vs *AF3* post-MD simulation

Table S6. Distances between HTH motifs in MabR structural models

Structural model	Distance (Å)[a]
RX C-MabR dimer	34.1
AF2 dimer 1	34.6
AF2 dimer 2	34.6
AF2 tetramer	34.5
AF3 dimer	97.7
AF3 tetramer	34.7

[a] The distance between the HTH motifs in the dimeric structures is measured as the distance between the Ca atoms of Arg378 of each monomer.

Figure S1. The four constructs used in the study and photographs of N/C-MabR crystals obtained

(a) MabR constructs

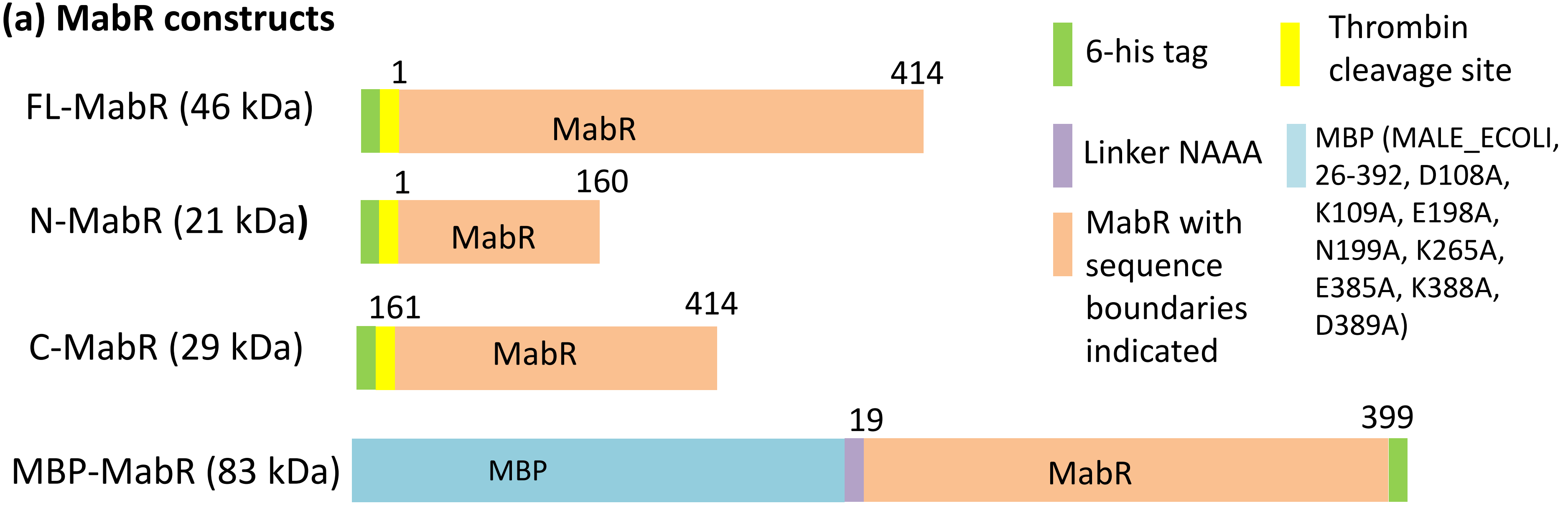

(b) MabR crystals

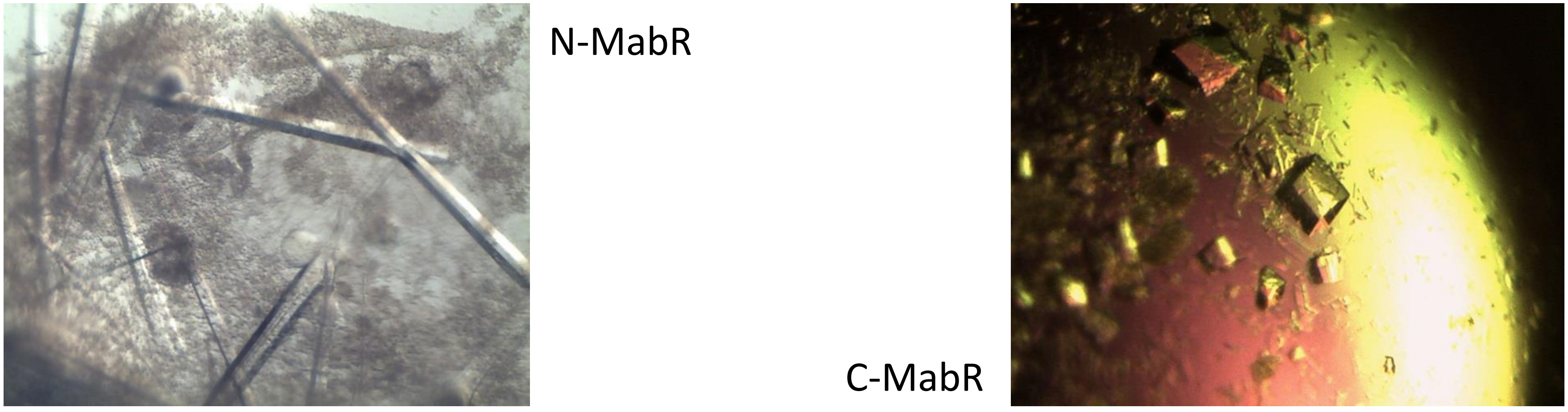

Figure S2. Mass spectrometry identification of the *E. coli* SlyD contaminant

Band

sp|P0A9K9|SLYD_ECOLI	FKBP-type peptidyl-prolyl cis-trans isomerase SlyD OS=Escherichia coli (strain K12) GN=slyD PE=1 SV=1	→ 192 spectres. Mass 20840
CON_sp|P00761|TRYP_PIG	Trypsin OS=Sus scrofa PE=1 SV=1	
sp|P0A6T5|GCH1_ECOLI	GTP cyclohydrolase 1 OS=Escherichia coli (strain K12) GN=folE PE=1 SV=2	→ 79 spectres. Mass 24815
sp|P0ACJ8|CRP_ECOLI	cAMP-activated global transcriptional regulator CRP OS=Escherichia coli (strain K12) GN=crp PE=1 SV=1	→ 43 spectres. Mass 23625
sp|P0A9A9|FUR_ECOLI	Ferric uptake regulation protein OS=Escherichia coli (strain K12) GN=fur PE=1 SV=1	→ 17 spectres. Mass 16784

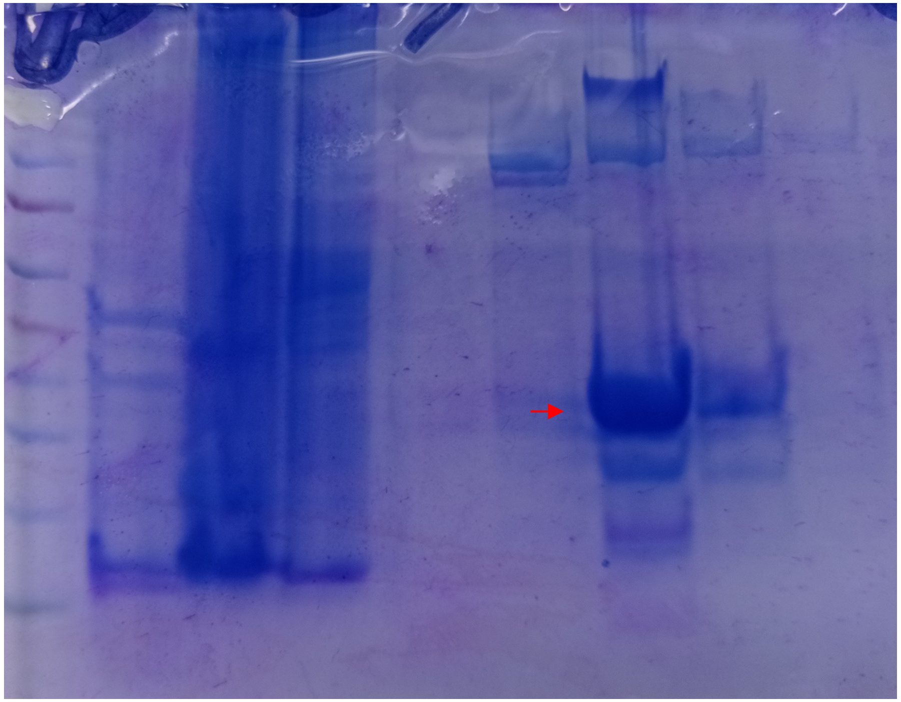

Figure S3. Global views of the phased electron density maps for N-MabR and C-MabR crystal structures

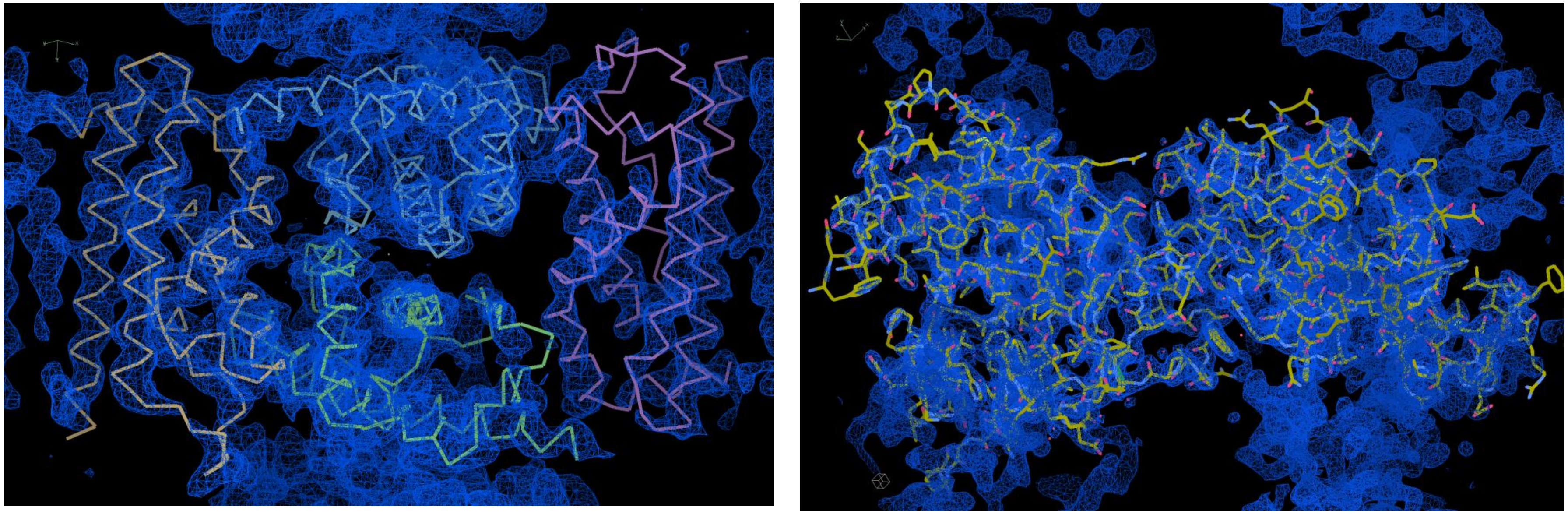

Global views of the phased 2Fo-Fc electron density maps (blue mesh) for the final models of N-MabR (left panel) and C-MabR (right panel). The maps are contoured using COOT at 1.5 rmsd and 2.0 rmsd above the average electron density for N-MabR and C-MabR, respectively. N-MabR is depicted as Cα trace, while C-MabR is shown as bond lines.

Figure S4. HPLC-gel filtration calibration curves

Gel filtration standards from Bio-Rad:
Bovine thyroglobulin (670 kDa), bovine γ-globulin (158 kDa), chicken ovalbumin (44 kDa), horse myoglobin (17 kDa) and vitamin B12 (1.35 kDa). Note that the vitamin B12 cannot be resolved using Protein-Pak 125 column.

A. Calibration curve for Xbridge Protein BEH SEC column

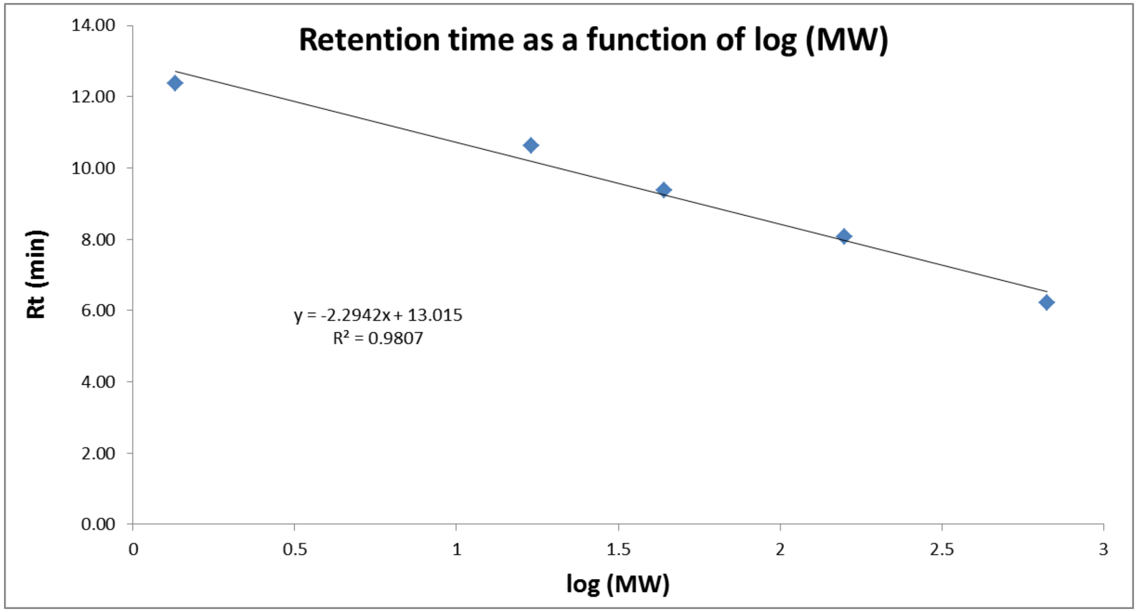

B. Calibration curve for Protein-Pak 125 column

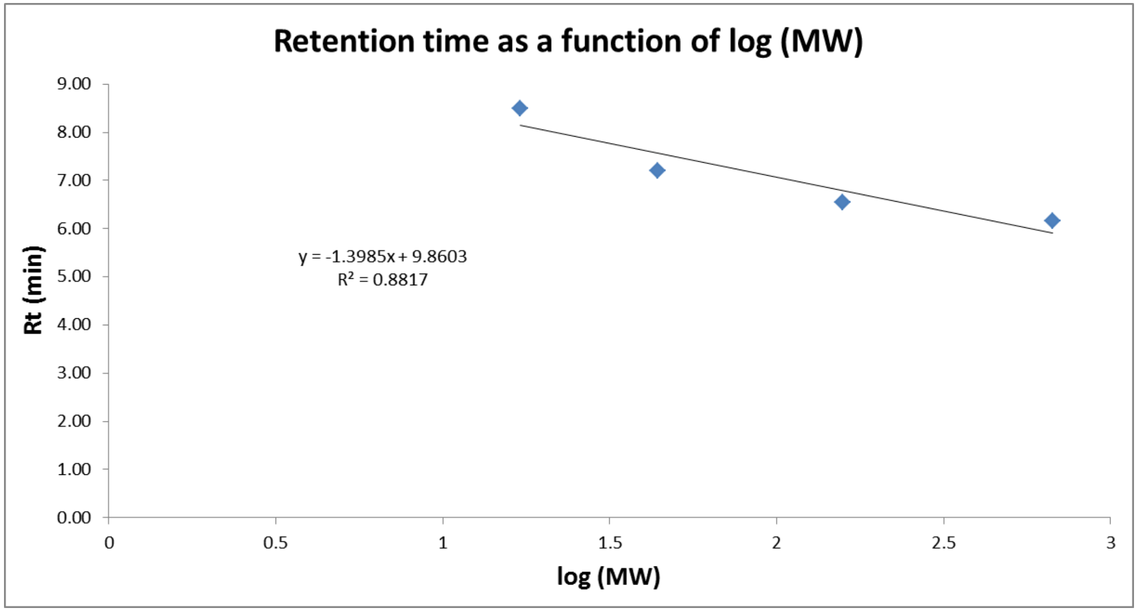

Figure S5. PISA interface analysis of the N-MabR crystal structure

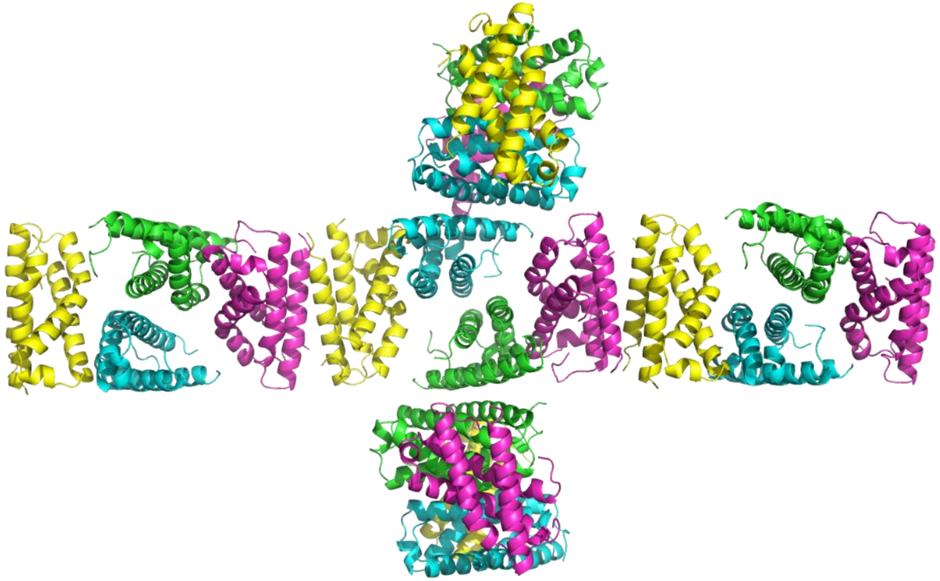

N-MabR crystal packing

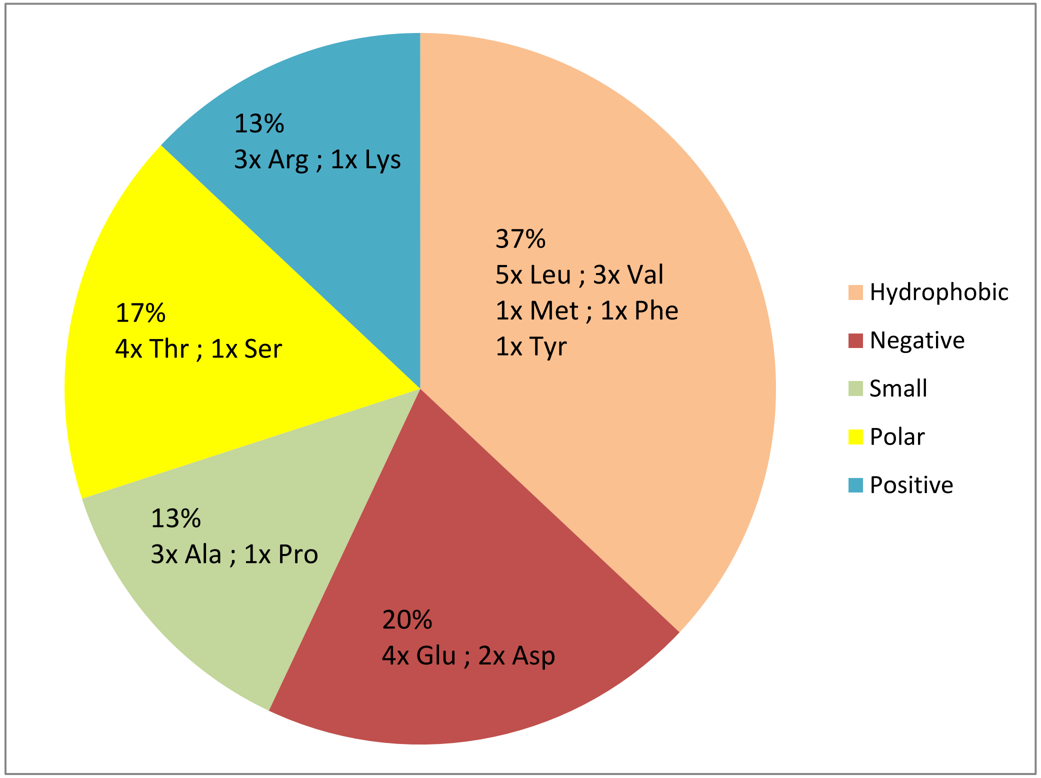

Interfacing residue content

Chain1	N_{res}	surf	Chain2	Symmetry op	N_{res}	Surf	inter	ΔG	ΔG p-value	N_{HB}	N_{SB}	CSS
A	29	8097	A	-x-1,y,-z	20	8097	1145	-20.7	0.079	6	8	1
C	27	7616	D	y-1,x,-z-1/4	31	7994	1048	-23.1	0.045	1	7	1
B	30	7636	B	X,-y,-z-1/2	30	7636	1203	-15.3	0.379	8	6	1

Chain1/Chain2: the two chains from which the interface was examined; N_{res}: number of residues participating in the interface; surf: surface accessible area of the chain in Å^2; Symmetry op: the symmetry operation that could be applied to the chain2 to build the respective interface; inter: interface area in Å^2, calculated as difference in total accessible surface areas of isolated and interfacing chains divided by two; ΔG: estimated solvation free energy gain upon formation of the interface, in kcal/Mol; ΔG p-value: the p-value of the solvation energy gain that is a measure of interface specificity, showing how surprising, in energy terms, the interface is. A value of p-value>0.5 means that the interface is likely to be an artefact of crystal packing, while p-value<0.5 suggests that the interface can be interaction-specific. The lower the p-value, the more likely the interface is, with le limiting case of p-value=0 means that such interface is a truly unique spot on the protein surface; N_{HB}/N_{SB}: number of potential hydrogen bonds/salt bridges across the interface; CSS: Complexation Significance Score, which indicates how significant for assembly formation is the interface, ranged from 0 (no significance) to 1 (most significant interface).

Figure S6. Schematic 2D representation of interactions at the N-MabR dimeric interfaces

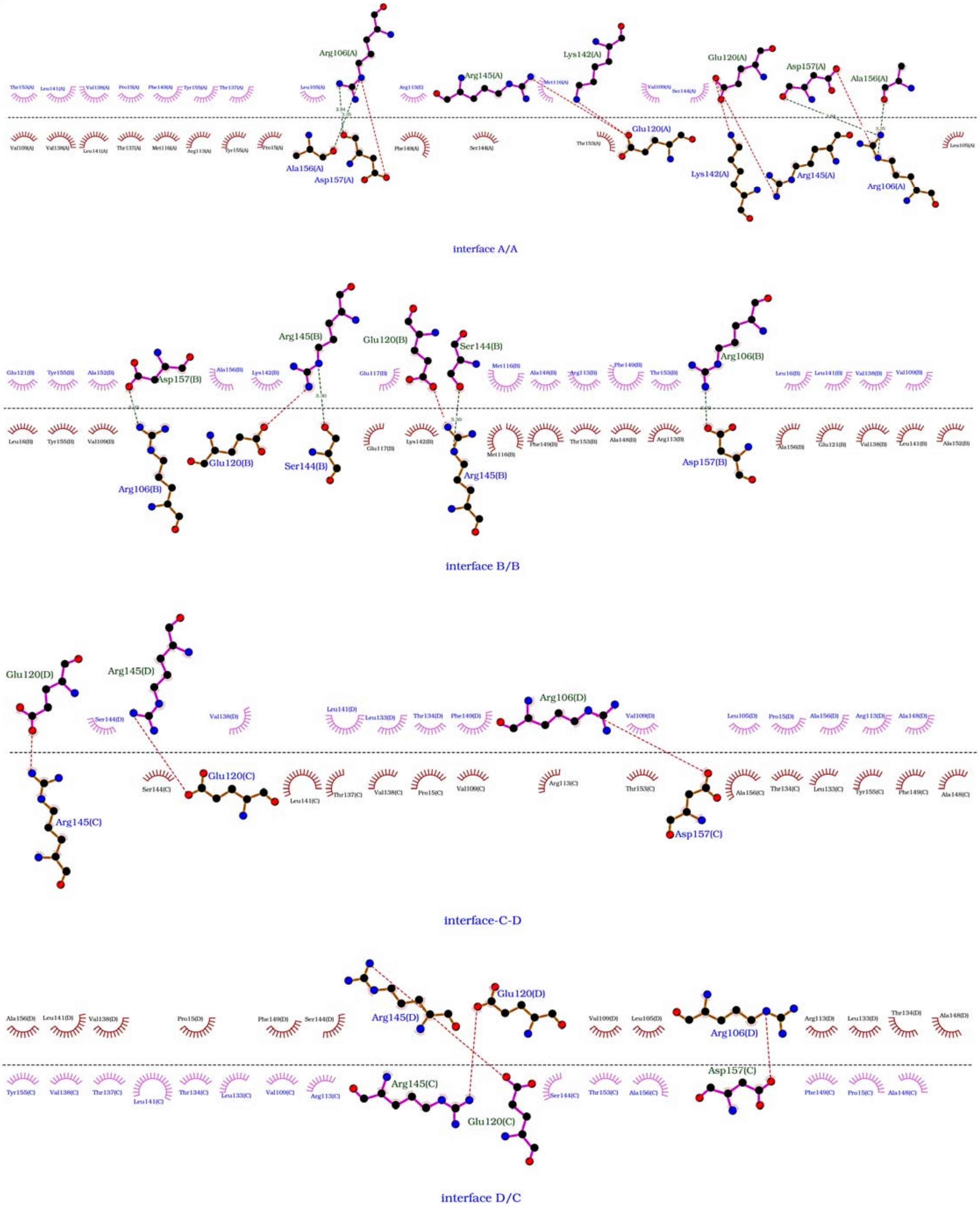

Plot 2D of the interactions across the N-MabR dimeric interfaces observed in the crystal structure. The horizontal dashed lines represent the protein-protein dimeric interfaces. Green dashed lines represent hydrogen bonds with its length indicated, red dashed lines are salt bridges and brick-red spoked arcs indicated hydrophobic contacts. Black, blue, and red spheres are carbon, nitrogen, and oxygen atoms, respectively. The image was produced with DimPlot from the program LigPlot+ 2.2.8 (Laskowski R.A., Swindells M.B., 2021, LigPlot+: multiple ligand-protein interaction diagrams for drug discovery. *J. Chem. Inf. Model.* 51, 2778-2786, DOI: 10.1021/ci200227u).

Figure S7. Structural comparison of N-MabR with two heme globin sensor domains: non-conservation of histidines essential in the chelation of iron

Globin domain of *Geobacter sulfurreducens* globin-coupled sensor (PDB id 2w31)

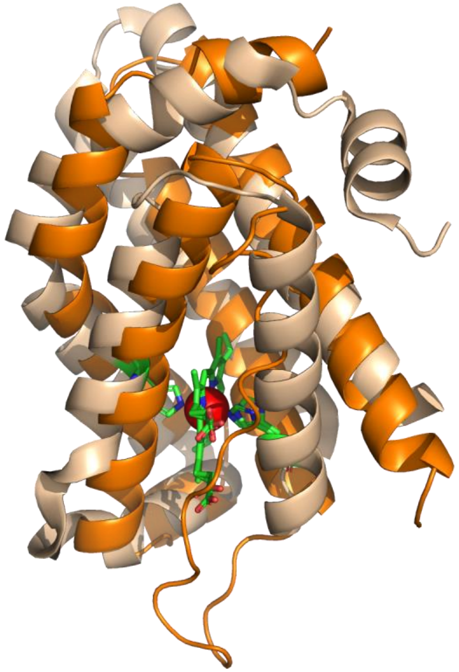

Rmsd 2.9 Å (#Cα 152)

Globin domain of *Escherichia coli* DosC (PDB id 4zvb)

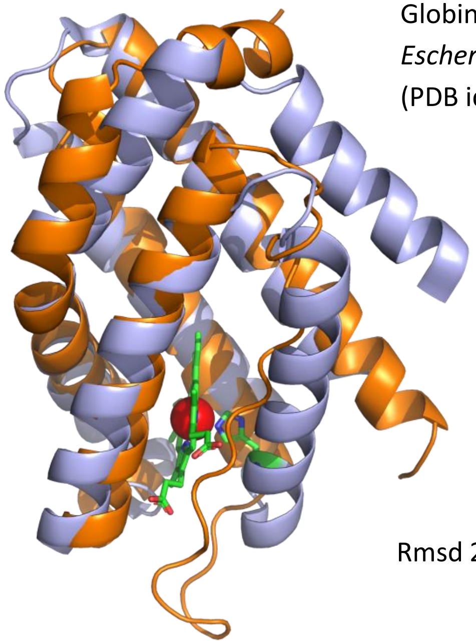

Rmsd 2.7 Å (#Cα 150)

```
DSSP   -----------LHHHHHHHLLHHHHHHHHHHHHHHHHHHHHHHHHhLHHHHHL-LHHH-h
N-MabR -----------SPLELLDTVPDSLLRRLKQYSGRLATEAVSAMQErLPFFADL-EASQ-r 47
2W31   mltmqeikahyRFTDEDAELLGSLFPLAETNKERLADQFYDYLLG-IPETAEFlKEDLvl 59
DSSP   lllhhhhhhhhLLLHHHHHHHHHHLHHHHHHLHHHHHHHHHHHHL-LHHHHHHhLLHHhh

DSSP   HHHHHHHHHHHHHHHHhhHLLLlLLLL--LLLLlllllllllllllHHHHHHHHHHHHHH
N-MabR ASVALVVQTAVVNFVEwmHDPHsDVGY--TAQAfelvpqdltrriaLRQTVDMVRVTMEF 105
2W31   QKLKQTHQDWFVSLFA--GSYD-NRYIhnLQKI-------ghahvrVGLNAHYVNVAMNV 109
DSSP   LLHHHHHHHHHHHLLL--LLLL-HHHHhhHHHH-------hhhhhhHLLLHHHHHHHHHH

DSSP   HHHHHHHLLL--------LHHHHHHHHHHHHHHHHHHHHhHHHHhhhll
N-MabR FEEVVPLLAR--------SEEQLTALTVGILKYSRDLAFtAATAyadaa 146
2W31   VRQFTLSIIQdnfpdpeeRRQRREAVEKILDINLDIMSA-SYRE----- 152
DSSP   HHHHHHHHHHhhlllhhhHHHHHHHHHHHHHHHHHHHHH-HHHL-----
```

```
DSSP   ----------lhHHHHHHLlHHHHHHHHHHHHHHHHHHHHHHHHH---LHHHHhlLHHHH
N-MabR ----------spLELLDTVpDSLLRRLKQYSGRLATEAVSAMQER---LPFFAdlEASQR 47
4ZVB   fkrmkdewtglvEQADPPIrAKAAEIAVAHAHYLSIEFYRIVRIDphaEEFLS--NEQVE 58
DSSP   lhhhhhhhhhhhHLLLHHHhHHHHHHHHHHHHHHHHHHHHHHHHLhhhHLLLL--LHHHH

DSSP   HHHHHHHHHHHHHHHHHhhllLLLLL--LLLL---LLLLllllllllLLHHhhHHHHHHH
N-MabR ASVALVVQTAVVNFVEWmhdpHSDVG--YTAQ---AFELvpqdltrrIALRqtVDMVRVT 102
4ZVB   RQLKSAMERWIINVLSA----QVDDVerLIQIqhtVAEV-----harIGIP--VEIVEMG 107
DSSP   HHHHHHHHHHHHHHHLL----LLLLHhhHHHHhhhHHHH-----hhhHLLL--HHHHHHH

DSSP   HHHHHHHHHHLL----LLHHHHHHHHHHHHHHHHHHHHHHHHHhhhll
N-MabR MEFFEEVVPLLA----RSEEQLTALTVGILKYSRDLAFTAATAyadaa 146
4ZVB   FRVLKKILYPVIfssdYSAAEKLQVYHFSINSIDIAMEVMTRA----- 150
DSSP   HHHHHHHHHHHHhlllLLHHHHHHHHHHHHHHHHHHHHHHHLL-----
```

Legend: the pairwise structural alignments were computed using DaliLite (rmsd and number of aligned Cα (#Cα) are given). The upper part of figure shows ribbon view of superimposed structures where N-MabR, *G. sufurreducens* globin (2W31) and *E. coli* DosC (4ZVB) are colored in orange, wheat and blue, respectively. Heme group found in the two globin sensors, as well as Fe-chelating histidines (His66 and His93 in 2W31 and His98 in 4ZVB), are depicted in stick representation with carbon atoms, oxygen and nitrogen in green, red and blue, respectively. Iron atom is show as red sphere. The lower part shows the structural alignment with secondary structures computed using DSSP (H/h: helix, E/e: strand, L/l: coil), where structure aligned residues are in uppercase. Fe-chelating histidines are highlighted.

Figure S8. Schematic 2D representation of interactions at the C-MabR dimeric interface

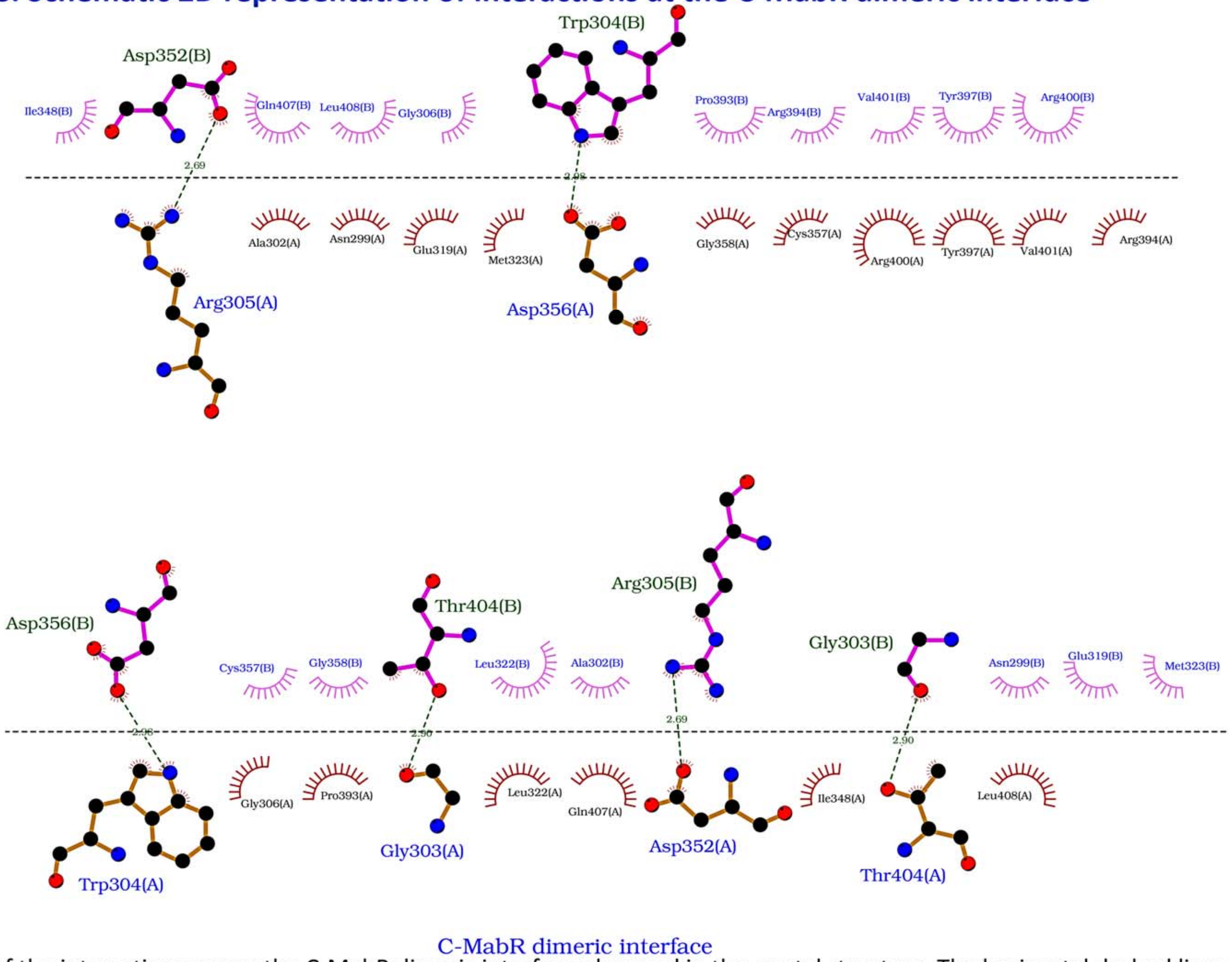

Plot 2D of the interactions across the C-MabR dimeric interface observed in the crystal structure. The horizontal dashed lines represent the protein-protein dimeric interface. Green dashed lines represent hydrogran bonds with iots length indicated while brick-red spoked arcs indicates hydrophobic contacts. Black, blue, and red spheres are carbon, nitrogen, and oxygen atoms, respectively. The image was produced with DimPlot from the program LigPlot+ 2.2.8 (Laskowski R.A., Swindells M.B., 2011, LigPlot+: multiple ligand-protein interaction diagrams for drug discovery. *J. Chem. Inf. Model.* 51, 2778-2786. DOI: 10.1021/ci200227u.

Figure S9. Dynamics analysis: oligomerization states of MBP-MabR in response to protein concentration and time progression

(a) Relative percentages of oligomerization states as a function of MBP-MabR concentration

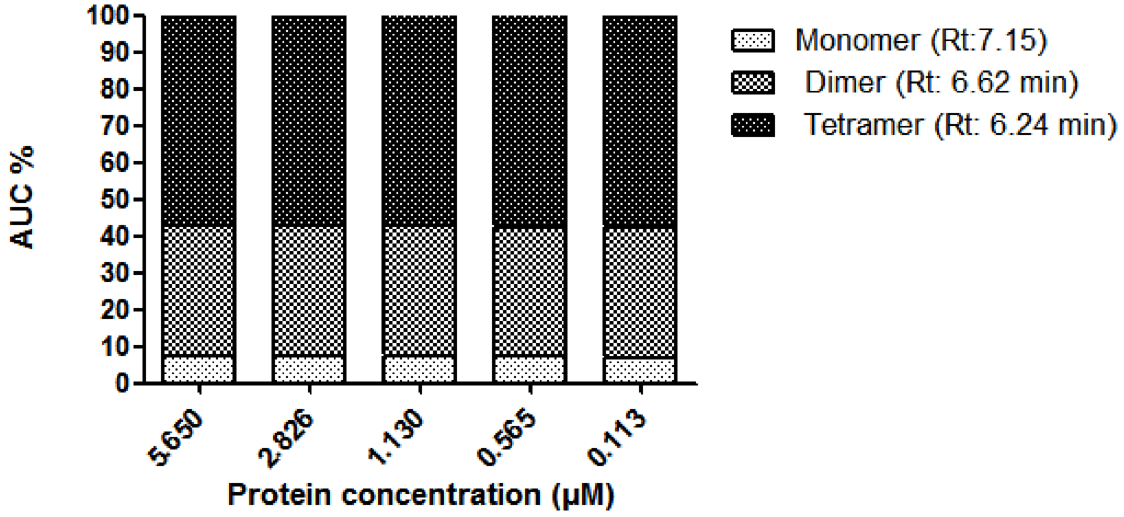

(b) Relative percentages after re-injection of the tetrameric peak

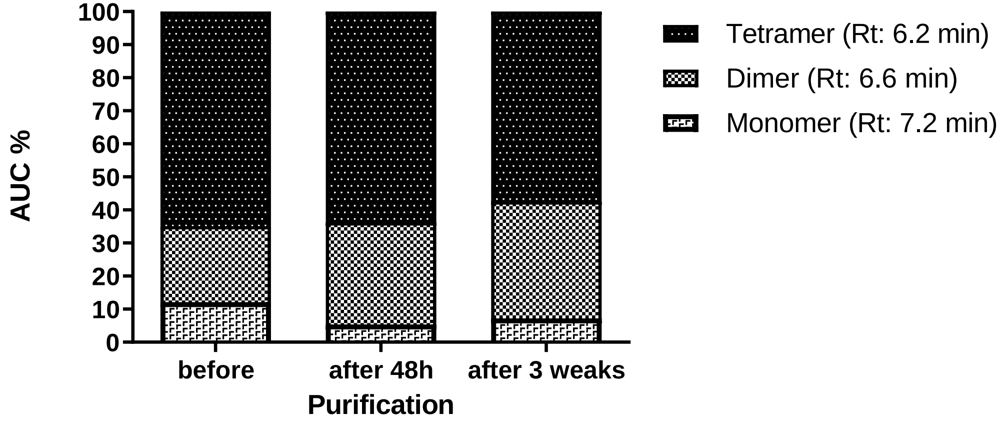

Figure S10. SEC-SAXS analysis of MBP-MabR

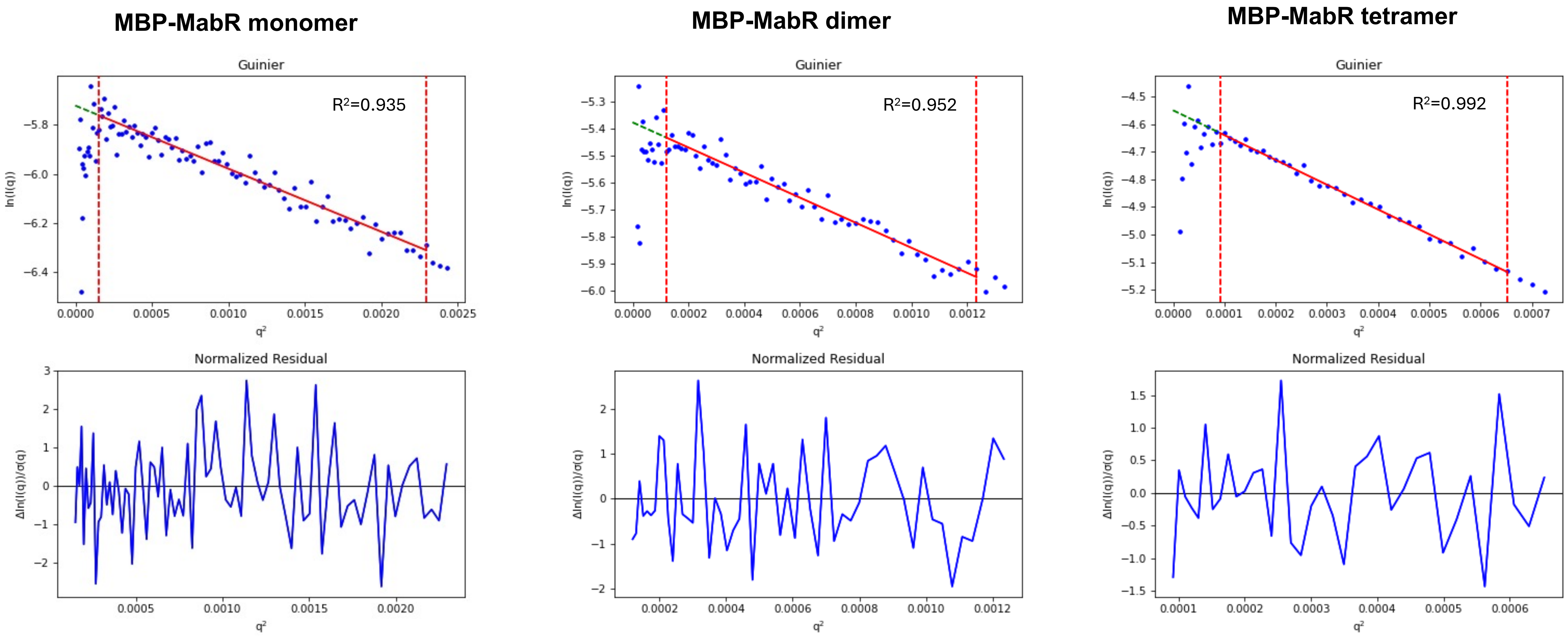

For the three selected frames in the SEC-SAXS profile, corresponding to monomer, dimer, and tetramer of MBP-MabR, the top plot shows the Guinier plot and the fit with the coefficient of determination R^2 indicated, while the bottom plot shows the residual of the fit. The plots were obtained from BioXTRAS RAW analysis (Hopkins, 2024, doi.org/10.1107/S1600576723011019).

Figure S11. ***AF2*** **MabR tetramer colored according to the confident score pLDDT**

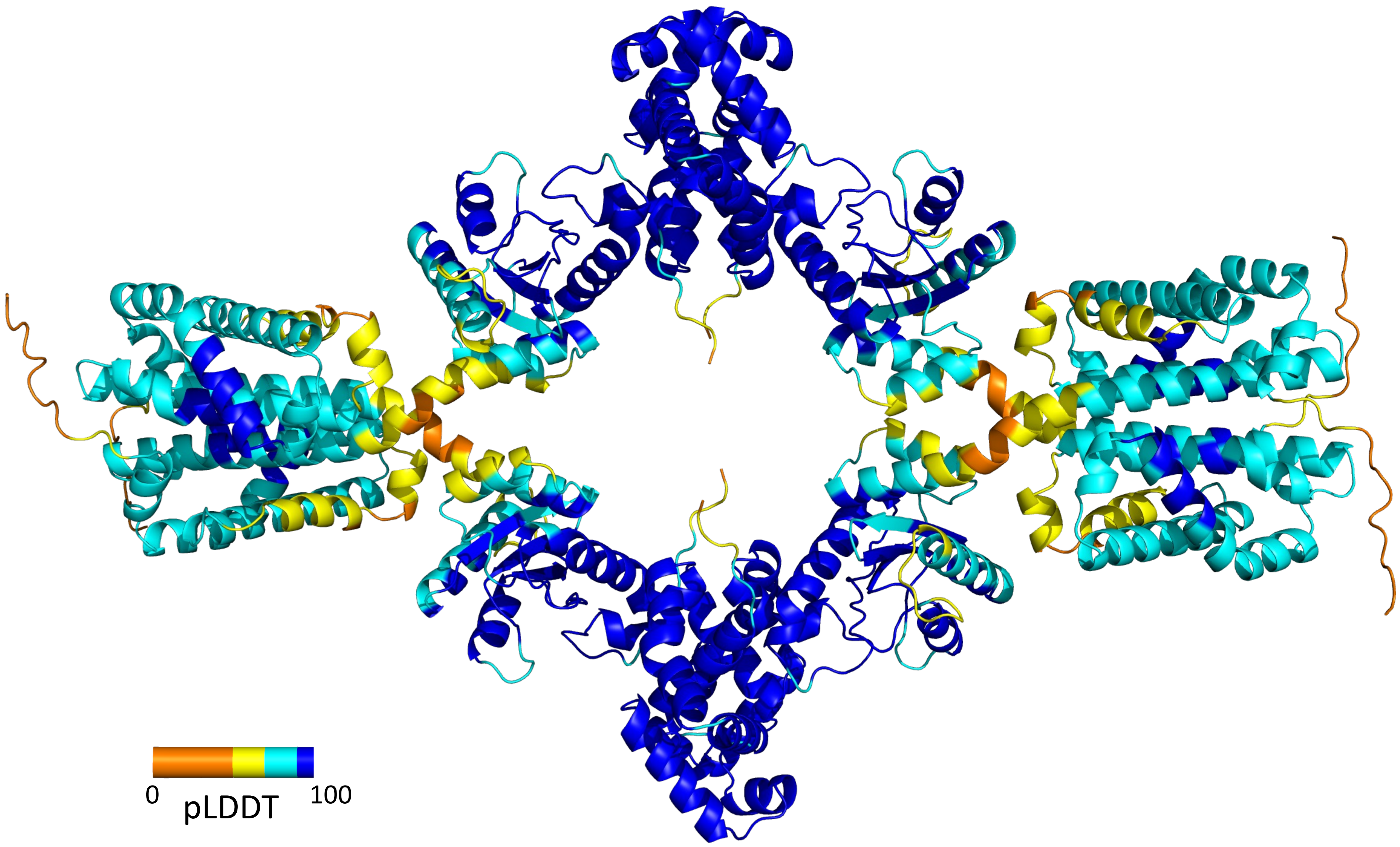

The per-residue confidence score, pLDDT (predicted Local Distance Difference Test), spans from 0 to 100 and gauges the confidence level of the predicted AF2 model in relation to the “ground truth” structure. pLDDT values are stratified into distinct confidence categories: very high confidence (pLDDT > 90; blue), high confidence (90 > pLDDT > 70: cyan, low confidence (70 > pLDDT > 50; yellow), and very low confidence (pLDDT < 50: orange).

Figure S12. MabR structural *AF3* models

MabR dimer

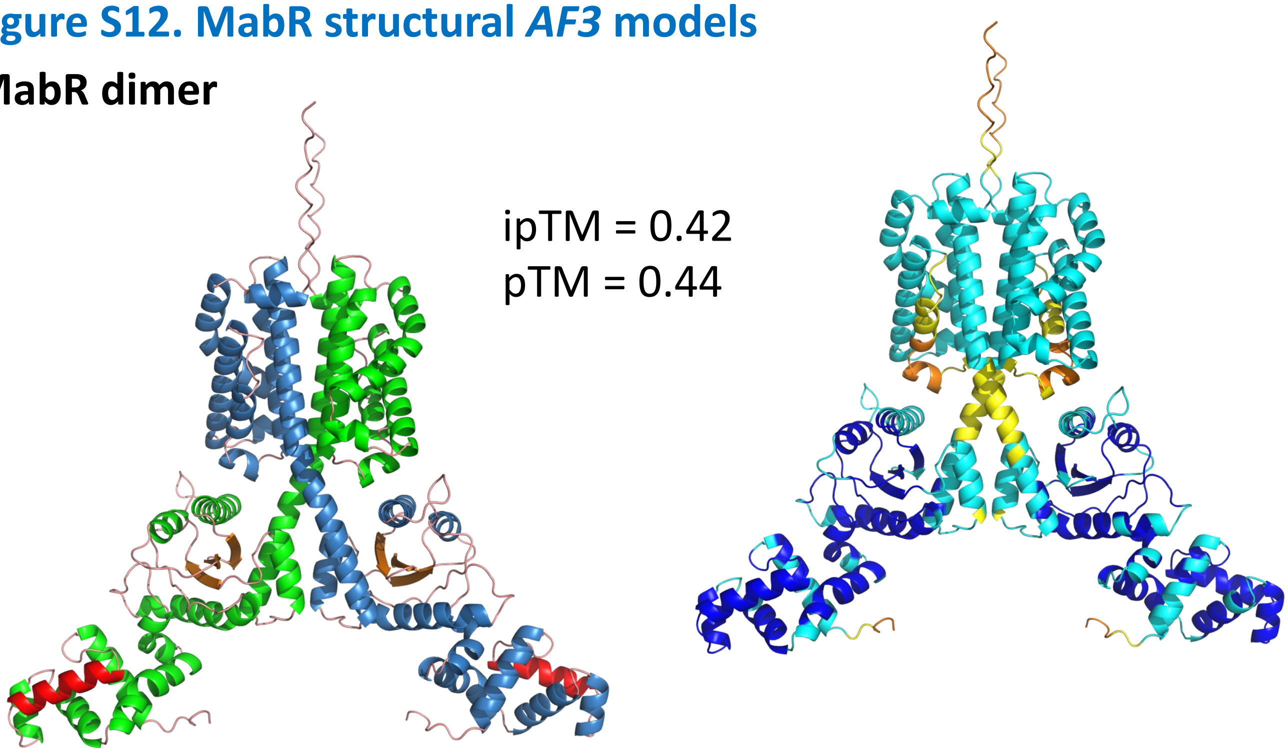

AF3 predicts a crossed dimeric organization for the MabR dimer, with the dimeric interface located at the N-terminal (see Table S4). The ribbon illustration on the left is colored by chain, with the recognition helices highlighted in red. The ribbon illustration on the right is colored according to the confidence scores (pLDDT). For details on this metrics, refer to Fig. S11.

MabR tetramer

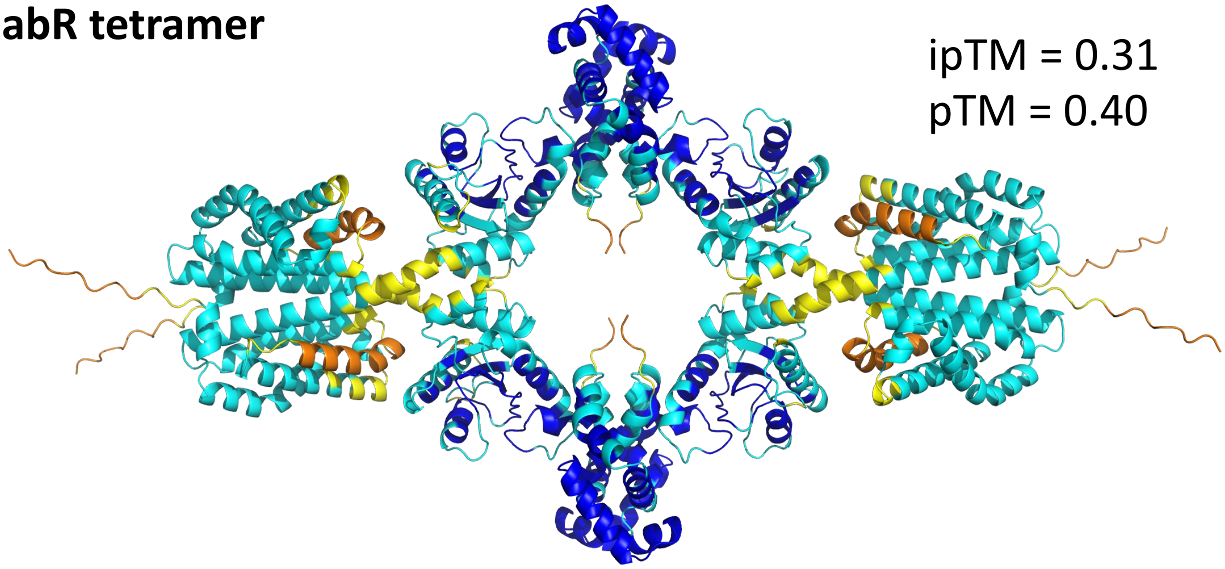

The predcited template modeling (pTM) score and the interface predicted template modeling (ipTM) score both measure the accuracy of the entire structure. A pTM score above 0.5 suggests that the overall predicted fold for the complex is likely similar to the true structure. ipTM scores higher than 0.8 indicate confident, high-quality predictions, while scores below 0.6 suggest a likely failed prediction. Scores between 0.6 and 0.8 fall into a gray zone where the accuracy of the predictions is uncertain.

Figure S13. AF2 models for MabR dimer

AF2 dimer 1

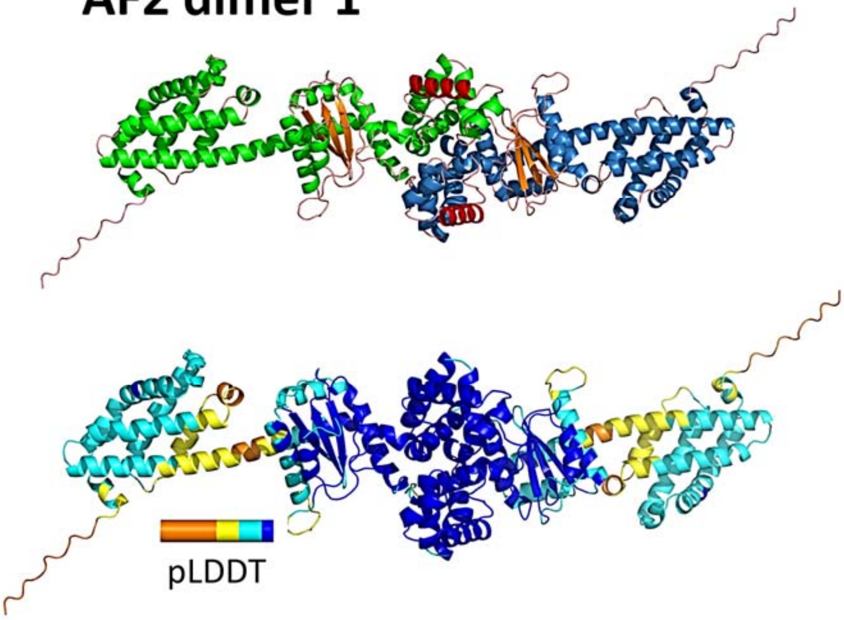

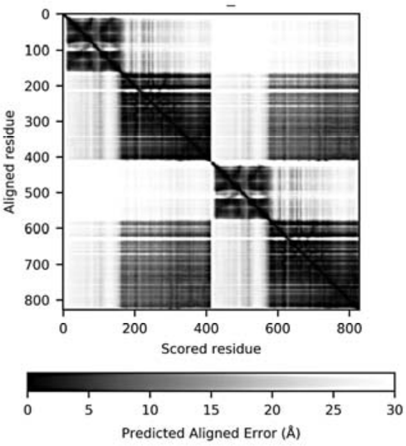

AF2 dimer 2

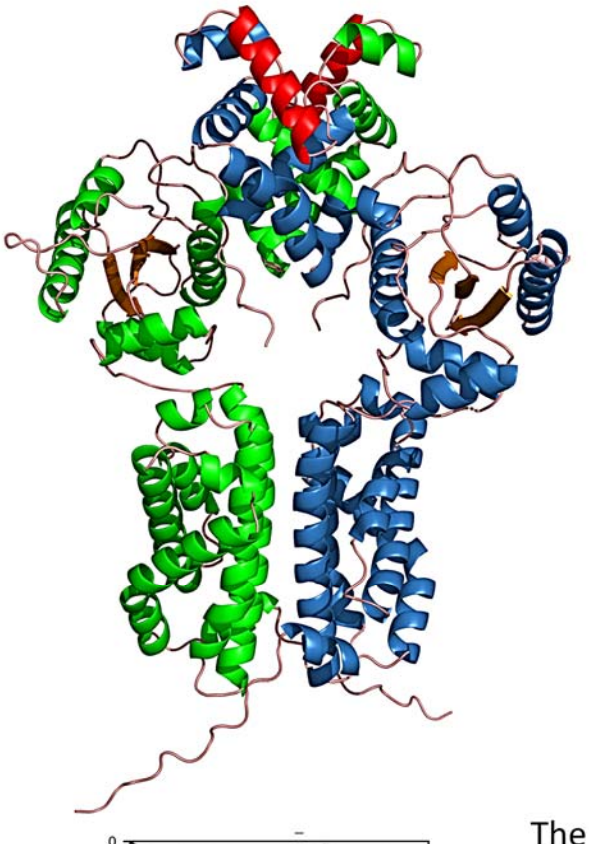

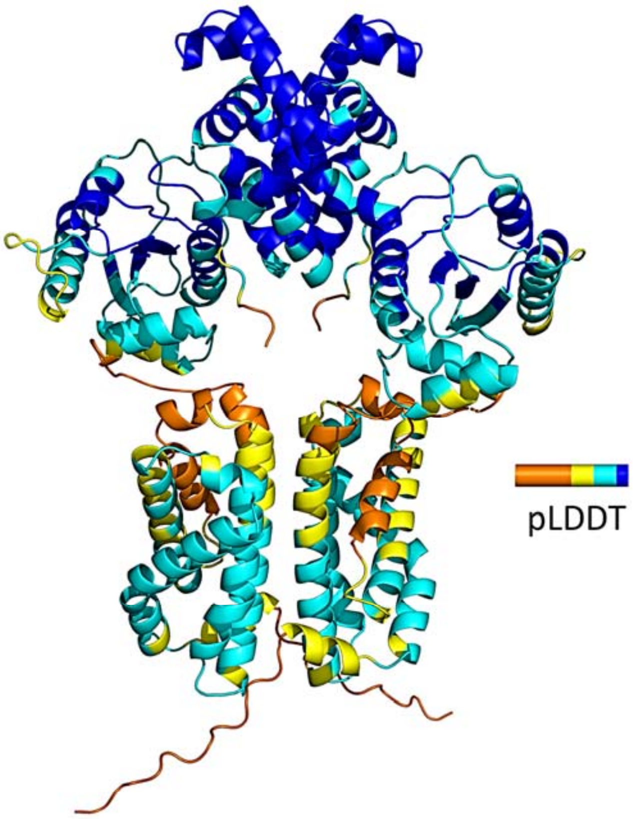

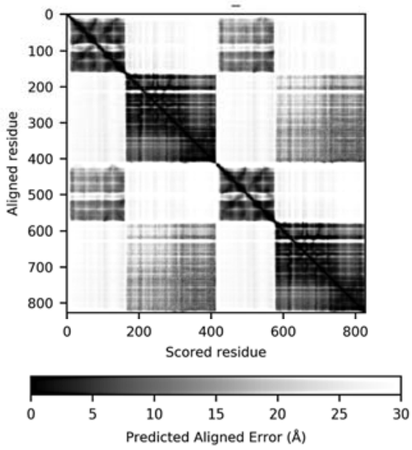

The two dimers are presented with their PAE diagram, and two ribbon illustrations: one colored by chain in where the recognition helices are in red and the second one colored according to their per-residue confidence scores pLDDT.

It is noteworthy that the pLDDT confidence scores of AF2 dimers are globally the same as in AF2 tetramer model (Figure S8), namely very high confidence for the C-MabR domains, high confidence for the N-MabR domains, and low confidence for the linker regions connecting these two domains.

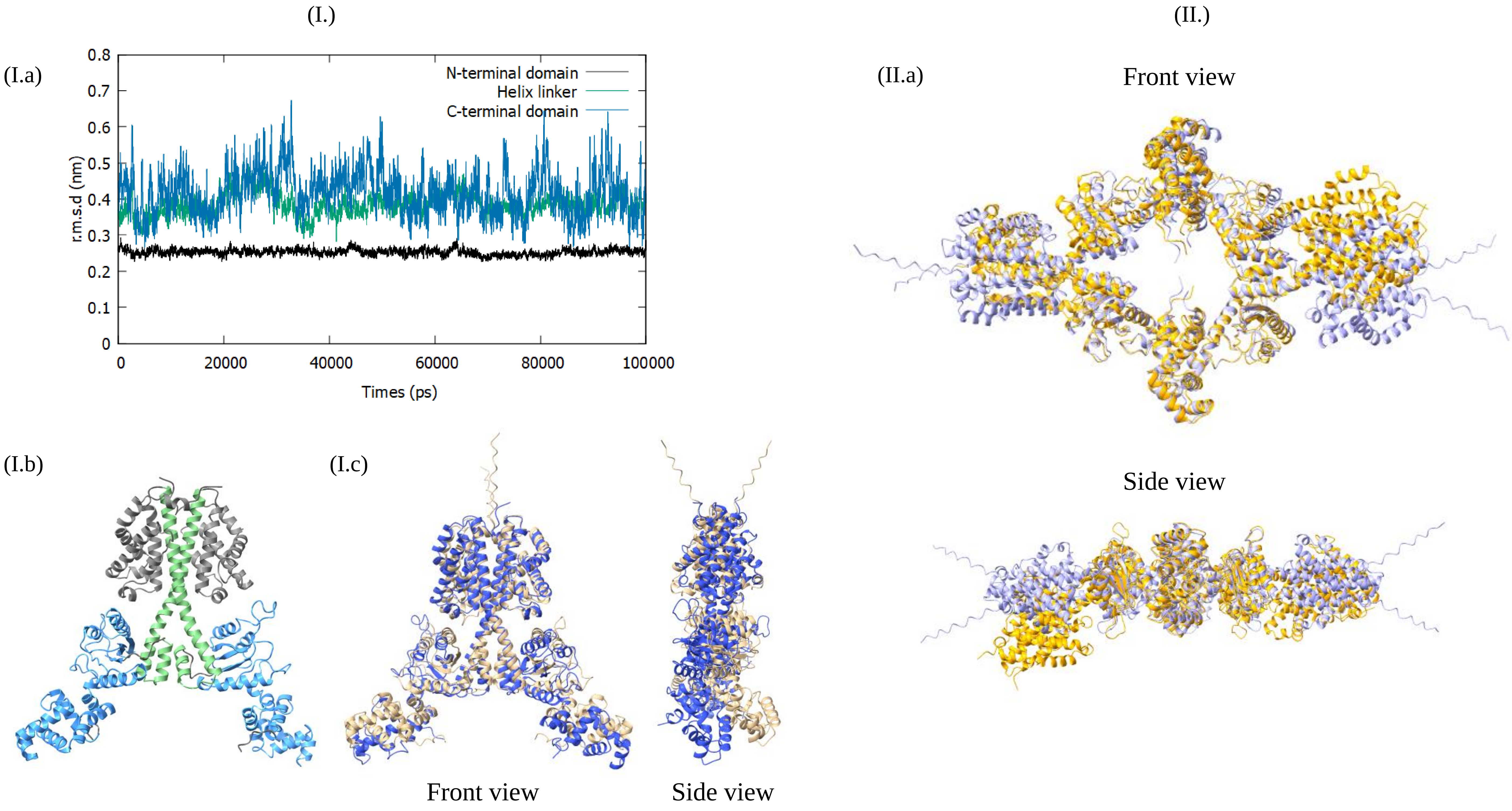

Fig. S14: Comparison of the initial *AF3* structure with the resulting structure after MD for the dimer (I) and the tetramer (II). (I.a) rmsd (in nm) calculated by GROMACS 2024 for the N-terminal domain (residues 15-126), helix linker (residues 130-193), and C-terminal domain (residues 201-408). Limits of domain were defined based on the first and the last residue involved in a secondary structure, as identified by DSSP. The rmsd shows stability in the N-terminal domain, while the linker and C-terminal domains exhibit relative flexibility. (I.b) Domain representation for DM analyses, with the N-terminal domain in grey, the helix linker in green, and the C-terminal domain in blue. (I.c) Structure superimposition between the initial AF3 model (in gold) and the N-terminal domain of the dimer after 100 ns of MD production (in blue). (II.a) Structure superimposition between the initial AF3 model (in lilac) and the C-terminal domain of the tetramer after 100 ns of MD production (in orange). The N-terminal domains show relative flexibility.

Figure S15. Fitting between experimental SAXS data and theoretical scattering profiles from AF2 models

MBP-MabR tetramer

χ2 = 2.803

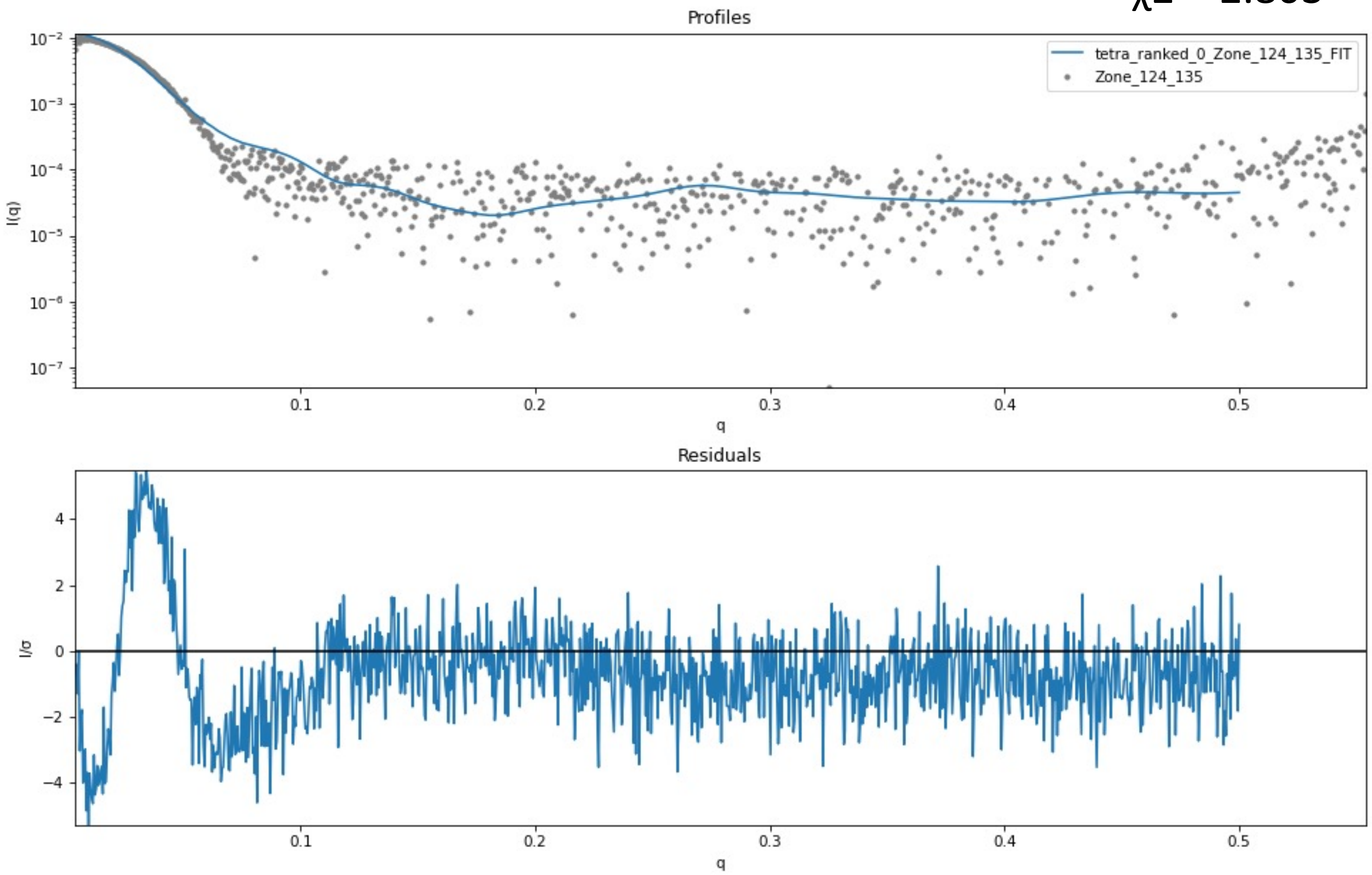

AF2 model

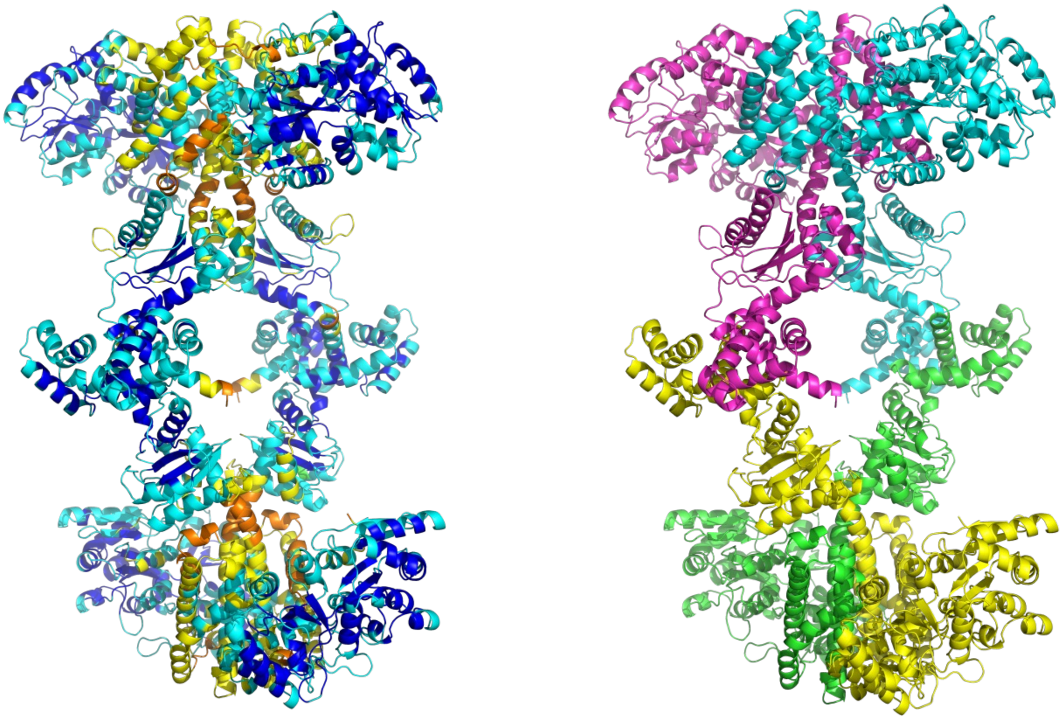

MBP-MabR dimer

χ2 = 1.399

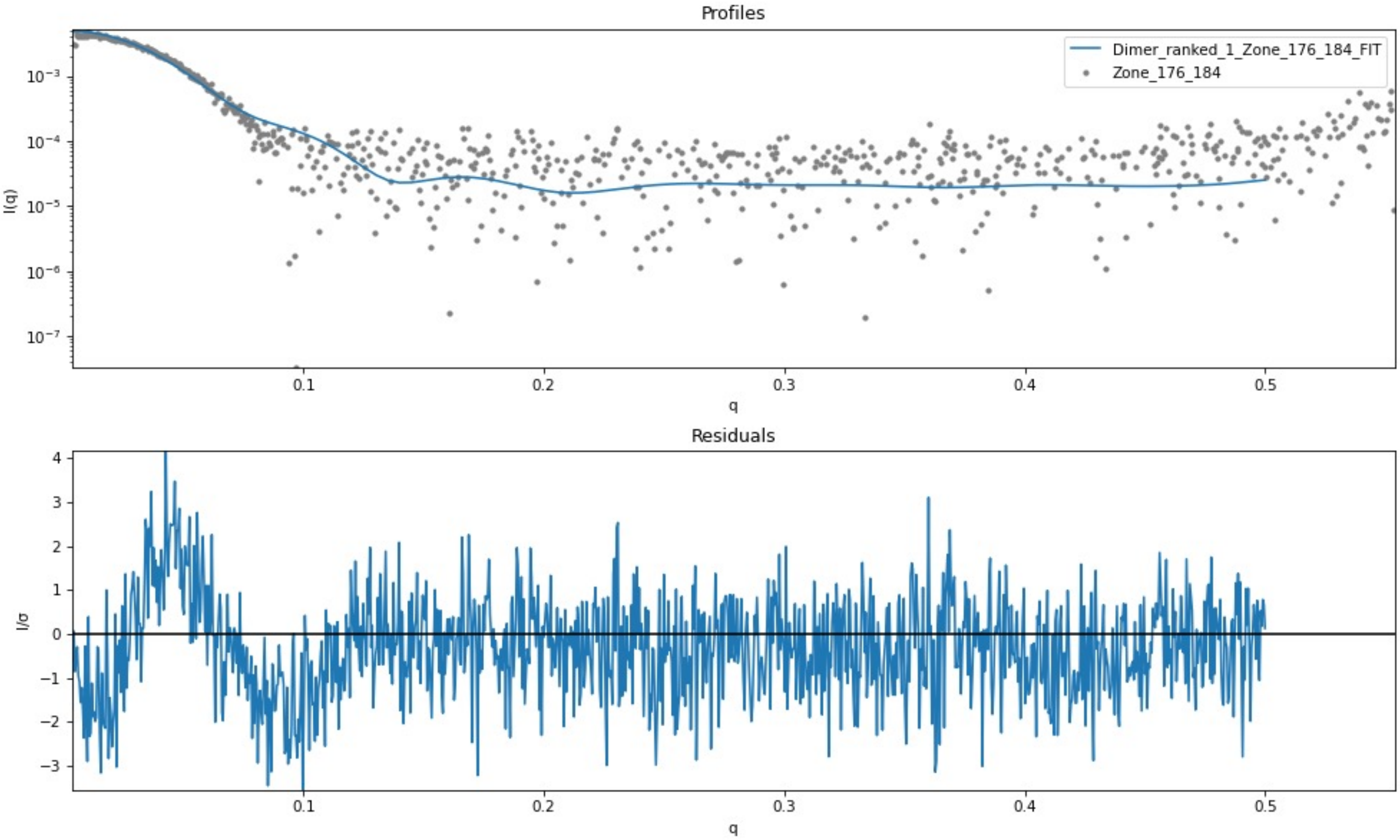

AF2 model

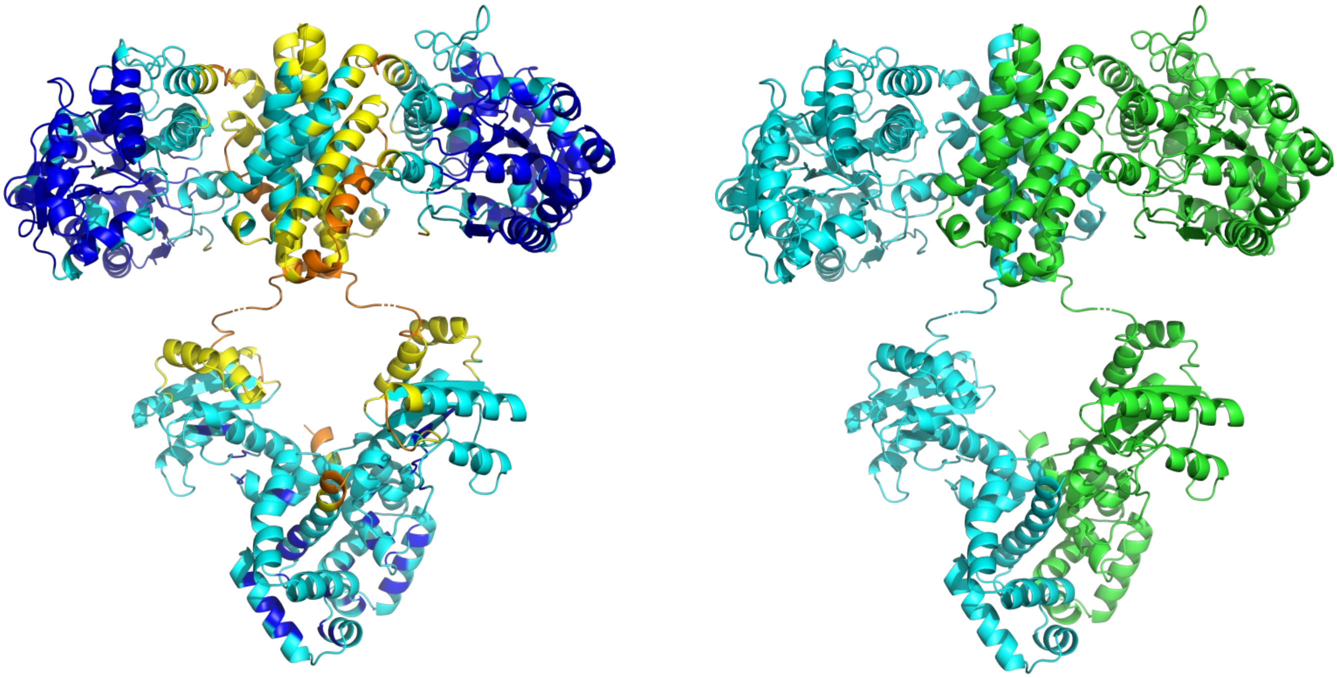

MBP-MabR monomer

χ2 = 2.280

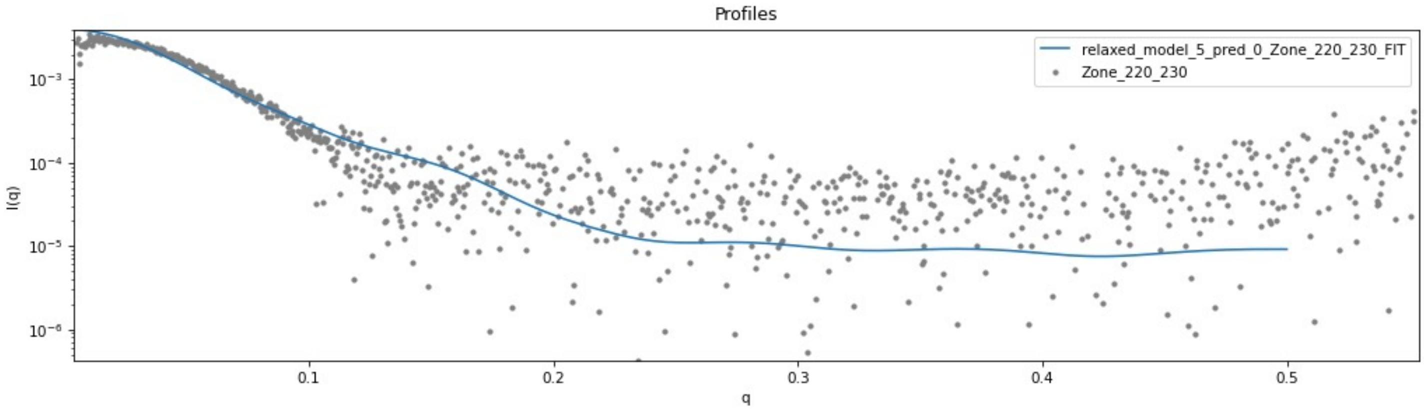

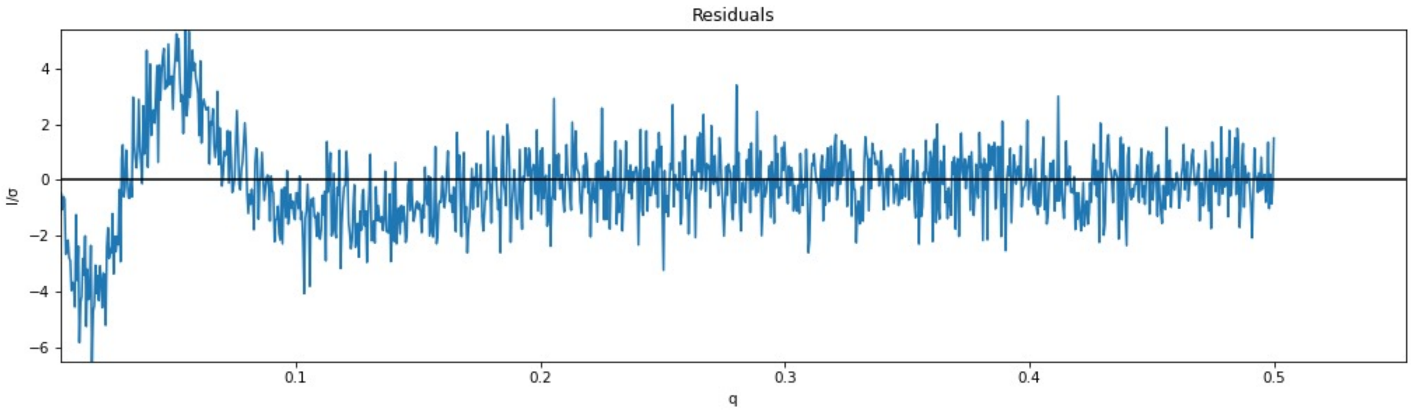

AF2 model

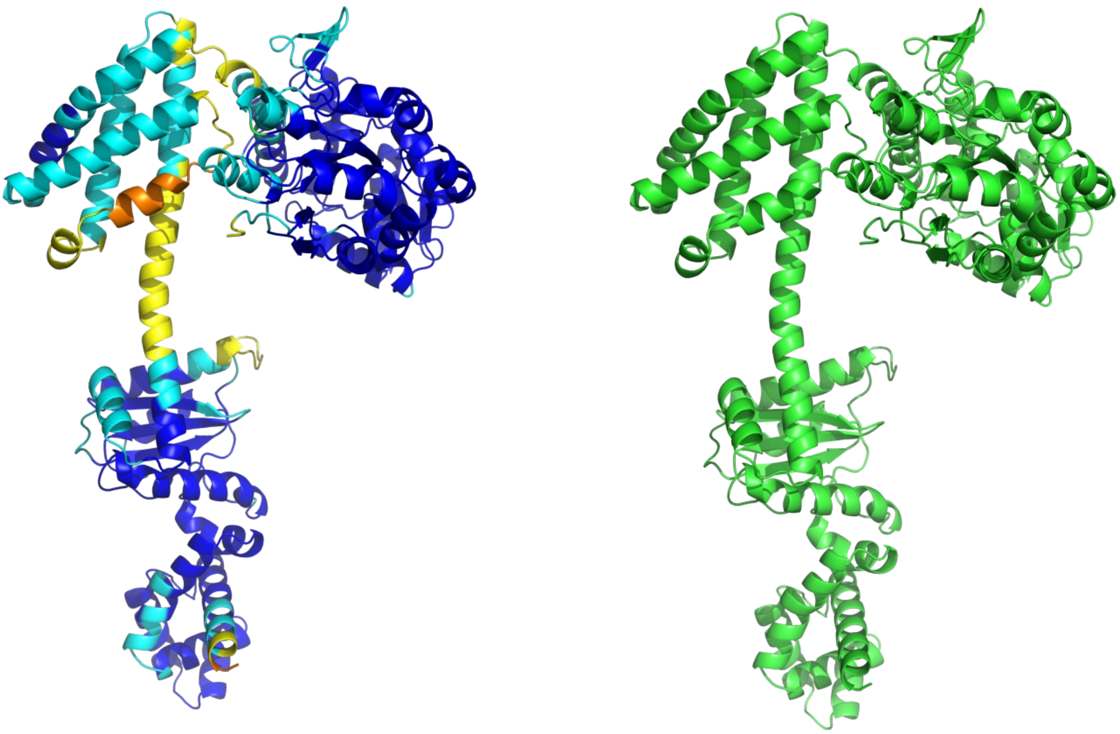

Figure S16. Isothermal analysis of the TSA curve for C-MabR/DNA complex

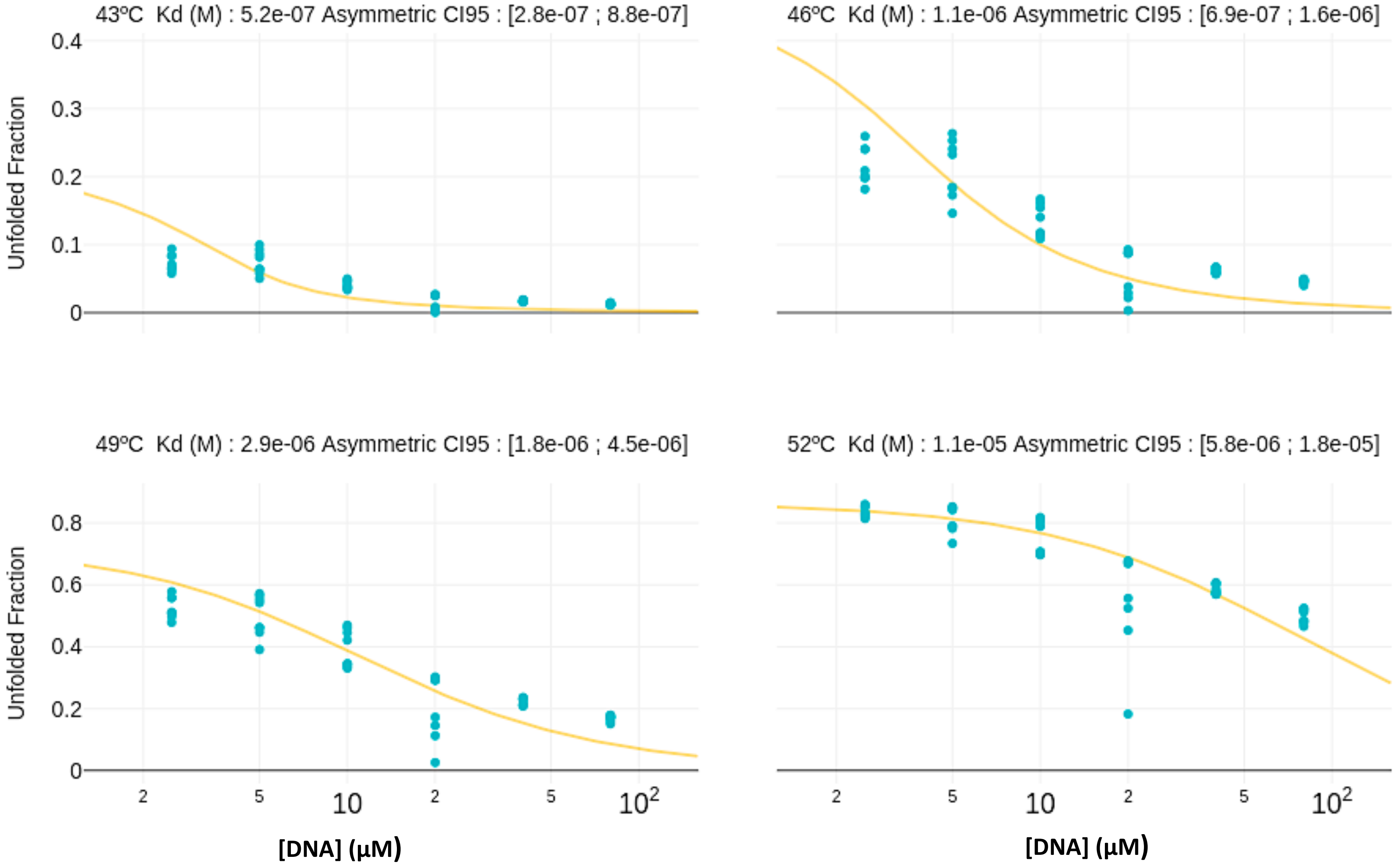

The melting curves of C-MabR (Figure 6d) were analyzed by isothermal approach implemented in FoldAffinity tool, allowing estimation of the binding affinity between C-MabR and the 23-bp double-stranded DNA, i.e., ~1 μM at 46°C and ~3 μM at 49°C.

Fig. S17. ITC analysis of MBP-MabR binding to DNA

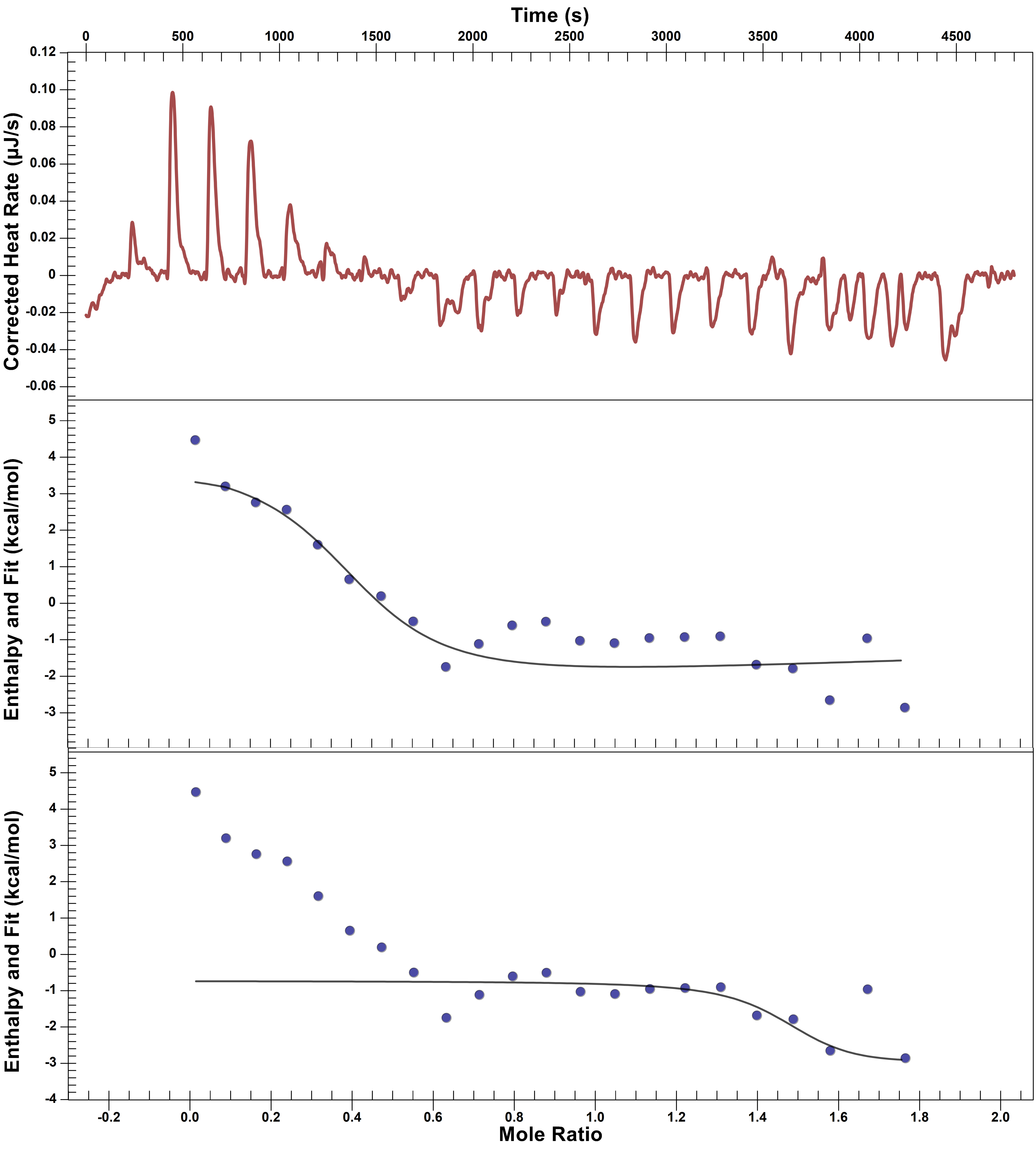

Supplement: Multimedia component 1 [file mmc1.pdf]
