# Supplementary material for: Domain architecture of the Mycobacterium tuberculosis MabR (Rv2242), a member of the PucR transcription factor family
Source: Heliyon. 2024 Nov 16;10(22):e40494. doi: 10.1016/j.heliyon.2024.e40494 (PMC11617747; doi:10.1016/j.heliyon.2024.e40494)
Supplement: Multimedia component 2 [file mmc2.docx]

**Supplemental Information**

**Table S1**. MabR domain boundary predictions

**Table S2.** Summary of PISA interface analysis for the crystal structures of C-MabR and 3onq

**Table S3**. *Foldseek* result summary using the *AF2* MabR model

**Table S4**. Comparison between dimeric interfaces of crystal and *AlphaFold* structures

**Table S5.** Structural comparisons of MabR *AF* models following MD simulations

**Table S6.** Distances between HTH motifs in MabR structural models

**Figure S1**. The four constructs used and photographs of N/C-MabR crystals

**Figure S2.** Mass spectrometry identification of the *E. coli* SlyD contaminant

**Figure S3.** Global views of the phased electron density maps for N-MabR and C-MabR crystal structures

**Figure S4**. HPLC-gel filtration calibration curves

**Figure S5**. PISA interface analysis of the N-MabR crystal structure

**Figure S6**. Schematic 2D representation of interactions at the N-MabR dimeric interfaces

**Figure S7**. Structural comparison of N-MabR with two heme globin sensor domains: non-conservation of histidines essential in the chelation of iron

**Figure S8**. Schematic 2D representation of interactions at the C-MabR dimeric interface

**Figure S9**. Dynamics analysis: oligomerization states of MBP-MabR in response to protein concentration and time progression

**Figure S10.** SEC-SAXS analysis of MBP-MabR

**Figure S11.** *AF2* MabR tetramer colored according to the confident score pLDDT

**Figure S12.** MabR structural *AF3* models

**Figure S13**. *AF2* models for MabR dimer

**Figure S14.** Comparison of the initial *AF3* structure with the resulting structure after MD for the dimer (I) and the tetramer (II)

**Figure S15.** Fitting between experimental SAXS data and theoretical scattering profiles from *AF2* models

**Figure S16**. Isothermal analysis of the TSA curves for C-MabR/DNA complex

**Figure S17**. ITC analysis of MBP-MabR binding to DNA

**Video file S1**. MD simulation of the *AF3* dimer

**Video file S2**. MD simulation of the *AF3* tetramer
